# Integrative discovery and targeted proteomics elucidate the plasma exosomal landscape in thyroid disorders, with emphasis on papillary thyroid carcinoma complicating Hashimoto’s thyroiditis

**DOI:** 10.3389/fendo.2026.1778949

**Published:** 2026-05-25

**Authors:** Yawei Zhang, Xiaolong Ma, Wenjing Shi, Tianyi Dong, Zhen Wang, Mao Ding, Xingchen Shang, Xingsong Tian

**Affiliations:** 1 Department of Breast and Thyroid Surgery, Shandong Provincial Hospital Affiliated to Shandong First Medical University, Jinan, Shandong, China; 2 Department of Thyroid Surgery, Shandong Provincial Hospital Heze Hospital (Heze Municipal Hospital), Heze, Shandong, China; 3 Medical Integration and Practice Center, Cheeloo College of Medicine, Shandong University, Jinan, Shandong, China

**Keywords:** exosomes, Hashimoto’s thyroiditis (HT), papillary thyroid carcinoma (PTC), proteomics, SVEP1

## Abstract

**Introduction:**

Thyroid cancer represents the most prevalent malignancy of the endocrine system, with papillary thyroid carcinoma (PTC) accounting for approximately 90% of cases. Hashimoto’s thyroiditis (HT) is a common benign thyroid disorder, frequently associated with structural alterations in thyroid morphology. Evidence indicated that HT may be a risk factor for the development of PTC. Exosomes promote intercellular communication by transferring proteins, mRNAs, micro-RNAs (miRNAs), and lipids to recipient cells, rendering them potential sources of disease-specific biomarkers.

**Methods:**

This study employed an integrative approach, combining exosome isolation, proteomics, and bioinformatics to identify plasma-derived exosomal biomarkers associated with PTC in the context of HT. Plasma samples were obtained from three patients each with PTC, PTC associated with HT (PTC-W), HT, and healthy controls (HCs). Exosomal proteins were extracted and analyzed using label-free quantitative proteomics.

**Results:**

A total of 45 differentially expressed proteins (DEPs) were identified between PTC-W and HT. Functional annotation and enrichment analyses revealed that these DEPs were predominantly implicated in extracellular matrix (ECM)–receptor interactions and immune response pathways. Subsequent validation via parallel reaction monitoring (PRM) confirmed that SVEP1 and IGKV3-7 exhibited consistent and statistically significant expression levels across discovery and validation phases.

**Discussion:**

These results highlight SVEP1 and IGKV3-7 as promising plasma exosomal biomarkers for PTC in patients with HT, providing mechanistic insights into the progression from HT to PTC and potential utility for non-invasive clinical diagnosis.

## 1 Introduction

Thyroid cancer has become the most common malignancy of the endocrine system, in which approximately 90% of cases are diagnosed with papillary thyroid carcinoma (PTC) (1). Hashimoto thyroiditis (HT) is the most common autoimmune disease, and studies mainly demonstrated that HT could be a risk factor for PTC. The chronic inflammation in HT may lead to reactive changes in stromal cells, potentially causing cell damage, thereby promoting tumor development (2) Although high-frequency ultrasound images and puncture cytology examination are clinically available, identifying high-risk populations for PTC among HT patients remains challenging. Therefore, it is essential to explore biomarkers for the early diagnosis of PTC complicating HT.

Exosomes are vesicles with a diameter of 30–150 nanometers (3) that can mediate intercellular communication. Donor cells can transfer exogenous substances, such as proteins, mRNAs, microRNAs (miRNAs), and lipids to recipient cells via exosomes (4). Current research on exosomes in thyroid diseases primarily concentrates on exosomal miRNA. A previous study has demonstrated that miR-16-2-3p, miR-223-5p, and miR-130a-3p (5) play a notable role in the diagnosis of PTC. The expression level of miR-21-5p in PTC patients is related to angiogenesis, promoting vascular formation, increasing tumor cell permeability, and enhancing cancer cell proliferation and the formation of a pre-metastatic niche (6). However, research on the role of plasma exosomal proteins in thyroid diseases is relatively limited, and they may serve as novel diagnostic biomarkers. A recent systematic review confirmed the strong potential of extracellular vesicles(EV) as biomarkers for thyroid cancer but highlighted that specific EV-associated proteins for the differential diagnosis of PTC from HT have not yet been investigated (7).

In this study, exosomes were isolated and characterized from blood specimens of patients with PTC, PTC complicated with HT, HT, and healthy controls (HCs) to investigate the differential expression of exosomal proteins among these groups. To elucidate the potential pathogenic effects of exosomal proteins in different specimens, functional enrichment analysis and interaction network analysis were performed, and differential proteins were validated by parallel reaction monitoring (PRM).

## 2 Materials and methods

### 2.1 Patients and tissue specimens

The study cohort comprised 3 patients with PTC, 3 patients with PTC complicated with HT, 3 patients with HT, and 3 HCs recruited from Shandong Provincial Hospital Affiliated to Shandong First Medical University (Jinan, China). The study protocol was approved by the Ethics Committee of Shandong Provincial Hospital Affiliated to Shandong First Medical University, and written informed consent was obtained from all participants.Inclusion criteria for all participants were age 18–80 years, regardless of sex. Specific criteria for each group were as follows: (1) PTC group: definitive pathological diagnosis of PTC. (2) PTC-W group: definitive pathological diagnosis of PTC and definitive laboratory or pathological diagnosis of HT. (3) HT group: definitive laboratory or pathological diagnosis of HT. (4) HC group: no personal or family history of thyroid disease; no history of any autoimmune disease; normal serum levels of thyroid function markers (FT3, FT4, TSH) and thyroid autoantibodies (TgAb, TPOAb, TRAb) within the reference ranges; and no suspicious thyroid nodules (TI-RADS ≥ 3), diffuse thyroid disease, or structural abnormalities on thyroid ultrasound.Exclusion criteria were: (1) presence of other autoimmune diseases (e.g., systemic lupus erythematosus, rheumatoid arthritis, Sjögren’s syndrome, IgA nephropathy, nephrotic syndrome), ruled out by medical history and physical examination; (2) presence of other malignancies or diseases affecting thyroid function; (3) acute or chronic infections;- severe organ dysfunction (heart, liver, or kidney); (4) pregnancy or lactation. Blood specimens were collected into K_2_EDTA vacuum tubes. To obtain plasma, samples were centrifuged at 3,000 × g for 15 min at 4 °C. After centrifugation, the supernatant was carefully transferred to a new tube and stored at −80 °C until further use.

### 2.2 Plasma exosome isolation and identification

For exosome isolation, a starting plasma volume of 6–12 mL per sample was used. Frozen plasma specimens were rapidly thawed at 37 °C and subjected to sequential gradient centrifugation. The samples were first centrifuged at 2000 × g for 30 min at 4 °C, and the supernatant was then collected. This was followed by centrifugation at 10000 × g for 45 min at 4 °C, after which the supernatant was filtered through a 0.45 μm filter membrane to obtain the filtrate. The filtrate was thereafter ultracentrifuged at 100000 × g for 70 min at 4 °C. The supernatant was carefully removed, and the resulting precipitate was resuspended in 10 mL of pre-cooled 1× phosphate-buffered saline. Using an ultracentrifugation rotor, the suspension was subjected to another round of ultracentrifugation under the same conditions (100000 × g,70 min, 4 °C). After discarding the supernatant, the pellet was resuspended in 600 μL of pre-cooled 1× PBS to obtain the exosome suspension.

For transmission electron microscopy (TEM) observation, 10 μL of the exosome sample was placed onto a copper grid and allowed to settle for 1 min. The excess fluid was gently removed with filter paper. Negative staining was then performed by adding 10 μL of uranyl acetate solution onto the grid for 1 min, followed by removal of the excess stain with filter paper. The grid was air-dried at room temperature for several minutes. Images were acquired using a transmission electron microscope operating at 100 kV. For exosome particle size analysis, a 10 μL aliquot of the exosome sample was diluted with PBS and analyzed using a NanoFCM N30E nano-flow cytometer (NanoFCM Co., Ltd., Xiamen, China). The instrument detects side-scattered light to determine particle size distribution and concentration. For surface marker characterization, exosomes were labeled with FITC-conjugated antibodies against CD9 (BD Biosciences, Cat 555371), CD63(BD Biosciences, Cat 557288), and CD81 (BD Biosciences, Cat 551108) and then analyzed on the same NanoFCM N30E instrument. Detailed procedures for labeling, washing, and data acquisition are provided in the **Supplementary Methods**.

### 2.3 Label-free quantitative proteomics

#### 2.3.1 Sample preparation

Protein concentration of the exosome samples was determined using the BCA (bicinchoninic acid) assay. Briefly, 20 μL of 10-fold diluted sample or BSA standard was mixed with 200 μL of BCA working solution in a 96-well plate, incubated at 37 °C for 30 min with shaking, and the absorbance was measured at 562 nm. For each sample, 100 μL of protein solution (corresponding to 51–241 μg total protein) was used for reduction and alkylation.

Dithiothreitol was added to the exosome sample to reach a final concentration of 5 mM, followed by incubation at 55 °C for 20 min. After cooling to room temperature, iodoacetamide (IAA) was added to a final concentration of 15 mM and the mixture was incubated in the dark for 30 min. Trypsin was dissolved in resuspension buffer to reach a concentration of 0.5 µg/µL and subsequently incubated at room temperature for 5 min. The trypsin solution was then thoroughly mixed with the sample, containing 0.1% formic acid (FA), 80% acetonitrile (ACN), and a trypsin-to-protein ratio of 1:50. After brief centrifugation, the mixture was incubated overnight at 37 °C with shaking at 1000 rpm. Subsequently, trifluoroacetic acid (TFA) solution was added to acidify the peptides. For the C18 cartridge, 1 mL of buffer C (ACN) and 1 mL of buffer A (0.1% FA in H_2_O, 2% ACN) were sequentially applied to activate and equilibrate the column. The sample supernatant was then loaded onto the cartridge. The column was washed twice with 1 mL of buffer A, followed by elution with 400 µL of buffer B (0.1% FA, 80% ACN). The eluate was collected in a new EP tube and vacuum-dried overnight at 4 °C to obtain the peptide sample.

#### 2.3.2 NanoLCMS/MS analysis

For each sample, 2μg of total peptides was separated and analyzed using a nanoUPLC (EASY-nLC1200) coupled to a Q Exactive HFX Orbitrap instrument (Thermo Fisher Scientific, Waltham, MA, USA) with a nanoelectrospray ion source. Separation was performed using a reversed-phase column (100 μm ID ×15 cm, Reprosil-Pur 120 C18AQ, 1.9 μm, Dr. Maisch). Mobile phases consisted of H_2_O with 0.1% formic acid (FA) and 2% acetonitrile (ACN) (phase A), and 80% ACN with 0.1% FA (phase B). Sample separation was performed using a 120-min gradient at a flow rate of 300 nL/min. Gradient B: 2–5% for 2 min, 5–22% for 88 min, 22–45% for 26 min, 45–95% for 2 min, and 95% for 2 min. Data-dependent acquisition (DDA) was carried out in profile and positive mode using an Orbitrap analyzer at a resolution of 120,000 (@200 m/z) and an m/z range of 350–1600 for MS1. For MS2, the resolution was set to 15,000 with a dynamic first mass. The automatic gain control (AGC) target for MS1 was set to 3E6 with a maximum ion transfer time (IT) of 50 ms, and 1E5 for MS2 with a maximum IT of 110 ms. The top 20 most intense ions were fragmented by HCD with a normalized collision energy (NCE) of 27%, and an isolation window of 1.2 m/z. The dynamic exclusion time window was set to 45 s, with single-charged peaks and peaks with charges exceeding 6 excluded from the DDA process.

#### 2.3.3 Database search and protein quantification

Raw MS files were processed using Proteome Discoverer software (Version 2.4.0.305) and the built-in Sequest HT search engine. MS spectra were searched against the species-level UniProt FASTA database (uniprot-Homo sapiens-2022-8.fasta), with carbamidomethylation (C) as a fixed modification and oxidation (M) and acetylation (protein N-term) as variable modifications. Trypsin was used as the protease, with a maximum of two missed cleavages allowed. The false discovery rate (FDR) was set to 0.01 at both the PSM and peptide levels. Peptide identification was performed with an initial precursor mass deviation of up to 10 ppm and a fragment mass deviation of 0.02 Da. Unique peptides and razor peptides were utilized for protein quantification, and the total peptide amount was used for normalization. All the other parameters were set to default.

### 2.4 Bioinformatics analysis

Principal component analysis (PCA) was conducted using SIMCA software (V16.0.2, Sartorius Stedim Data Analytics AB, Umea, Sweden). Differentially expressed proteins (DEPs) were identified by a fold change ≤ 0.83 or ≥ 1.2 and P < 0.05. Volcano plots, heatmaps, and a Venn diagram were generated to illustrate the distribution and overlap of DEPs across pairwise comparisons. Subsequently, subcellular localization prediction, eukaryotic orthologous group (KOG) classification, Gene Ontology (GO) enrichment analysis, Kyoto Encyclopedia of Genes and Genomes (KEGG) pathway analysis (https://www.kegg.jp/kegg/pathway.html), and protein-protein interaction (PPI) network analysis were performed on the DEPs (including both up-regulated and down-regulated).

### 2.5 PRM quantitative proteomics

#### 2.5.1 Sample preparation

Protein extracts from the same exosome specimens were collected and subjected to dithiothreitol reduction, IAA alkylation, and protein digestion with trypsin. Following sodium deoxycholate (SDC) cleanup and desalting using C18 spin columns, the supernatant was collected and vacuum-dried overnight at 4 °C to obtain peptide samples.

#### 2.5.2 NanoLC-MS/MS analysis

For each sample, 2 µg of total peptides was separated and analyzed using a nano-UPLC (EASY-nLC1200) coupled to a Q Exactive HFX Orbitrap instrument (Thermo Fisher Scientific) with a nano-electrospray ion source. Separation was performed using a reversed-phase column (100 mID ×15 cm, Reprosil-Pur 120 C18-AQ, 1.9 μm, Dr. Maisch). Mobile phases consisted of H_2_O with 0.1% FA and 2% ACN (phase A), and 80% ACN with 0.1% FA (phase B). Sample separation was performed using a 90-minute gradient at a flow rate of 300 nL/min. Gradient B: 2–5% for 2 min, 5–22% for 68 min, 22–45% for 16 min, 45–95% for 2 min, and 95% for 2 min. Parallel reaction monitoring (PRM) was conducted in centroid and positive mode using an Orbitrap analyzer at a resolution of 15,000 (@200 m/z) for MS2. The AGC target for MS2 was set to 1 × 10^5^. Predefined inclusion ions were fragmented by HCD with a NCE of 27% and an isolation window of 0.7 m/z.

#### 2.5.3 Data analysis

Raw files were converted to mzML format after peak picking at both MS1 and MS2 levels using Proteome Discoverer software (version 2.4.0.305) with vendor-specific algorithms. Database searches were performed using the MSFragger search engine (v3.1.1) against a species-specific FASTA database (uniprot-Homo sapiens-9606-2022-11.fasta), applying the recommended parameters for peptidomics analysis. The MSFragger search results were further processed using the Philosopher pipeline (v3.3.11).

## 3 Results

### 3.1 Baseline characteristics

The baseline demographic and thyroid-specific parameters of the four groups (PTC, PTC-W, HT, HC) are summarized in **Table 1**. Due to the small sample size (n=3 per group), formal statistical comparisons were not performed between groups.

**Table 1:** Baseline demographic and clinical characteristics of the study groups.

Characteristics	PTC (n=3)	PTC+HT (n=3)	HT (n=3)	HC (n=3)
Age (years)	40 (31-61)	48 (34-55)	53 (45-55)	41 (33,52)
Femal n (%)	2 (66.67)	2 (66.67)	3 (100)	2 (66.67)
BMI (Kg/m^2^)	22.8 (22.5-23.0)	23.2 (21.8-23.4)	23.1 (22.7-23.3)	23.2 (21.5-23.5)
FT3 (pmol/L)	4.01 (3.73-4.19)	4.41 (3.62-4.63)	3.9 (3.22-5.26)	4.35 (3.87-5.12)
FT4 (pmol/L)	11.80 (11.09-11.95)	11.70 (11.58-12.33)	12.6 (9.98-13.06)	15.83 (12.67-18.44)
TSH (uIU/mL)	1.58 (1.30-2.55)	3.76 (1.89-3.92)	2.36 (0.65-2.53)	1.55 (0.86-3.97)
TgAb (IU/mL)	13.90 (12.30-16.10)	275 (245-419)	32.5 (23.2-218)	18.73 (17.45-23.98)
TPOAb (IU/mL)	<9.0	86.8 (4.5-108)	77 (10.6-103)	<9.0
TRAb (IU/L)	0.87 (0.40-0.97)	0.4 (0.4-1.25)	<0.8	<0.8

Continuous variables are presented as median (range), categorical variables as n (%). Normal reference ranges: FT3 2.43–6.01 pmol/L, FT4 9.01–19.05 pmol/L, TSH 0.27–4.20 mIU/L, TgAb <115 IU/mL, TPOAb <34 IU/mL, TRAb 0–1.22 IU/L. For values below the detection limit (TRAb <0.8 IU/L, TPOAb <9.0 IU/mL), half the detection limit (0.4 IU/L and 4.5 IU/mL, respectively) was used for statistical calculation.

### 3.2 Exosome identification

**Figure 1:**
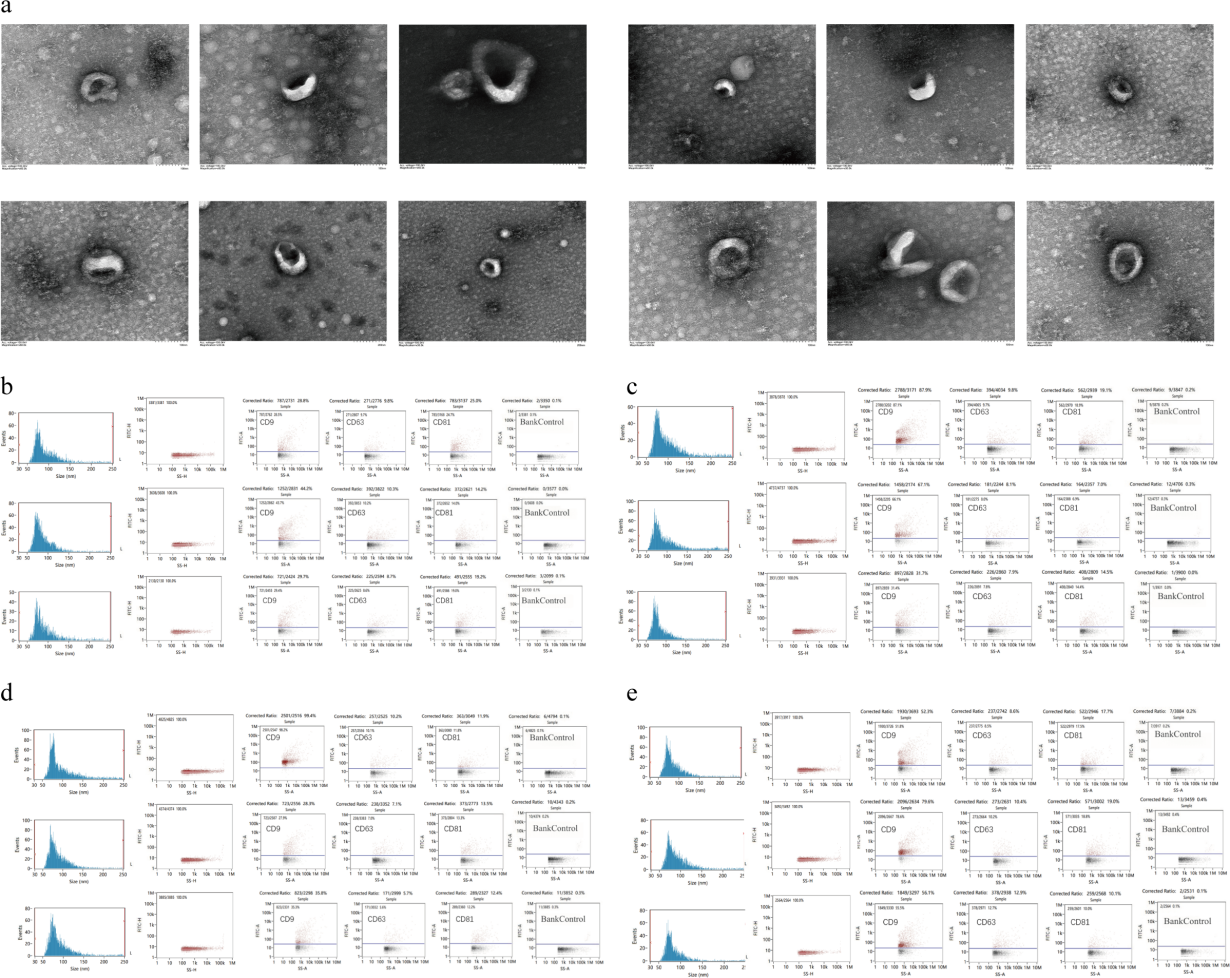
Characterization of plasma-derived exosomes. **(a)** TEM image showing the typical cup-shaped morphology of exosomes. Scale bar = 100 nm. **(b-e)** Nano-flow cytometry analysis of exosomes from PTC **(b)**, PTC-W **(c)**, HT **(d)**, and HC **(e)** groups, performed on a NanoFCM N30E instrument (NanoFCM Co., Ltd., Xiamen, China). For each group, the left panel shows the particle size distribution and concentration measured by side-scattered light (SSC). EV concentrations (particles/mL) were not significantly different among the four groups (one-way ANOVA, p>0.05). The right panel shows the positive expression rates of exosomal tetraspanin markers CD9, CD63, and CD81. These markers were stained separately using FITC-conjugated antibodies: FITC Mouse Anti-Human CD9 (BD Biosciences, Cat 555371), FITC Mouse Anti-Human CD63 (BD Biosciences, Cat 557288), and FITC Mouse Anti-Human CD81 (BD Biosciences, Cat 551108).

TEM revealed the presence of plasma-derived exosomes in all samples, with some exhibiting the characteristic “cup-shaped” morphology (**Figure 1a**). Particle size analysis indicated that the average diameter of exosomes ranged from 85 to 100 nm (**Figures 1b-e**). Fluorescent labeling combined with nanoscale flow cytometry demonstrated positive expression of three classical exosomal markers (CD9, CD63, and CD81) in the isolated exosomes (**Figures 1b-e**).

### 3.3 Plasma protein profile of thyroid disease

**Figure 2:**
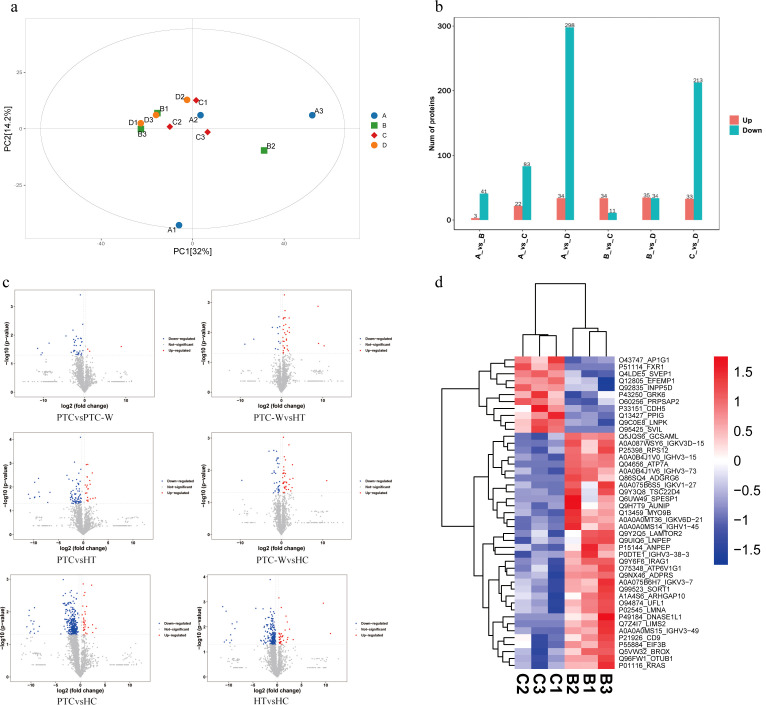
**(a)** PCA of identified proteins. PCA was performed using SIMCA software (V16.0.2, Sartorius Stedim Data Analytics AB, Umea, Sweden) after log transformation and mean-centering of the data. **(b)** Bar chart of the distribution of DEPs numbers in different comparison groups. **(c)** Volcano plotdepicts the DEPs in different groups. **(d)** Heatmap depicts the DEPs between PTC-W and HT. The group identifiers in the figure correspond to: A=PTC group, B=PTC-W group, C=HT group, D= HC group.

Plasma samples were subjected to label-free proteomic analysis. Raw data files were processed using Proteome Discoverer software (version 2.4.0.305, Thermo Fisher Scientific), along with the built-in Sequest HT search engine for database matching, resulting in the identification of 2,875 proteins and 23,354 peptides. Among them, 2,750 proteins were quantified using one or more unique peptides. PCA was performed on these 2,750 proteins (**Figure 2a**), followed by the assessment of protein expression profile differences across pairwise comparisons among PTC, PTC-W, HT, and HC. The distribution of DEPs across distinct comparative groups is illustrated in **Figure 2b**. DEPs (fold-change ≤ 0.83 or ≥ 1.2, p-value < 0.05) were visualized using volcano plots (**Figure 2c**). Hierarchical clustering was employed to generate a heatmap (**Figure 2d**) for DEPs between PTC-w and HT, revealing similar expression patterns within groups and distinct differences between groups. The DEPs were found to be primarily associated with extracellular matrix receptor interactions, protein translational modifications, and immune regulation.

### 3.4 Identification of plasma protein signatures in thyroid disease

To identify potential proteins eligible for the early detection of PTC, DEPs were analyzed through Venn diagram analysis, subcellular localization, KOG analysis, GO enrichment analysis, KEGG pathway analysis (8–10), and PPI network analysis.

**Figure 3:**
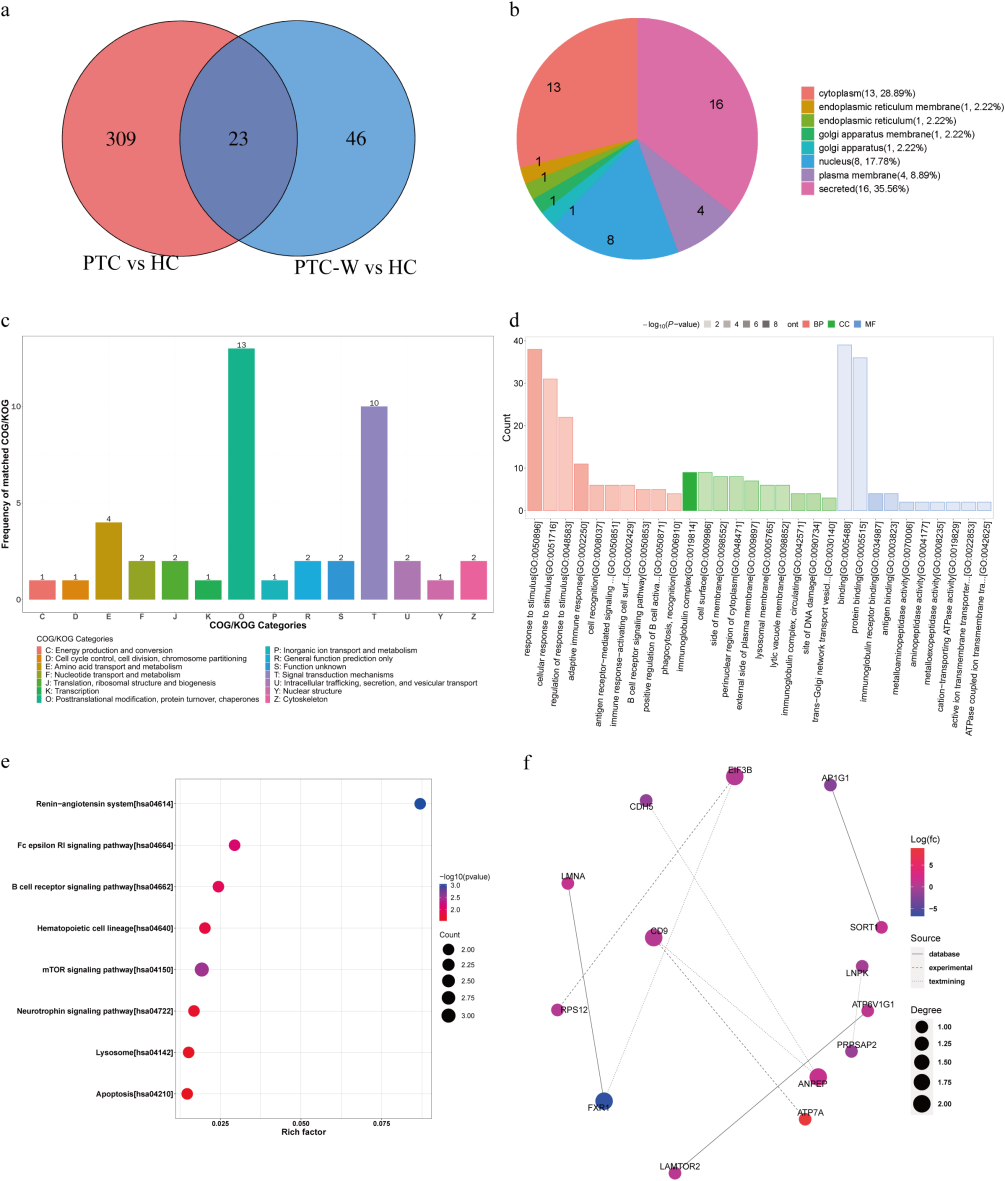
**(a)** Venn diagram showing the overlap of DEPs between the PTC vs HT and PTC-W vs HT comparisons. The numbers in each section indicate the count of DEPs unique to each comparison or shared between the two comparisons. **(b)** Subcellular localization analysis of DEPs identified in the PTC-W vs HT comparison. **(c)** KOG bar plot of DEPs identified in the PTC-W vs HT comparison. **(d)** GO term enrichment analysis of DEPs identified in the PTC-W vs HT comparison. **(e)** KEGG pathway enrichment analysis of DEPs identified in the PTC-W vs HT comparison. **(f)** PPI network of DEPs identified in the PTC-W vs HT comparison, constructed using the STRING database.

To visualize the overlap and specificity of DEPs between the two comparisons, a Venn diagram was constructed (**Figure 3a**). As shown, 23 DEPs were common to both comparisons, 309 DEPs were unique to the PTC versus HT comparison, and 46 DEPs were unique to the PTC-W versus HT comparison. The shared DEPs suggest common pathophysiological mechanisms between PTC and PTC-W relative to HT, whereas the unique DEPs in the PTC-W versus HT comparison highlight the specific molecular alterations associated with the coexistence of PTC and HT. DEPs in the PTC-W versus HT were predominantly localized to the secreted, cytoplasmic, and nuclear compartments (82.23%) based on subcellular localization analysis (**Figure 3b**). The KOG analysis revealed that these DEPs were primarily involved in posttranslational modification, protein turnover, chaperones, and signal transduction mechanisms (**Figure 3c**). The GO functional analysis further elucidated that the principal biological processes (BPs) were associated with these proteins. CD9 and IGKV3–7 were significantly enriched in immune-related responses, including response to stimulus, cellular response to stimulus, and regulation of response to stimulus. Regarding cellular components (CCs), the DEPs were mainly enriched in the immunoglobulin complex, cell surface, side of membrane, and perinuclear region of cytoplasm. Molecular function (MF) analysis indicated that the DEPs were primarily involved in binding and protein binding (**Figure 3d**). The KEGG enrichment analysis of DEPs revealed their predominant involvement in signaling pathways, such as the mTOR signaling pathway, Fc epsilon RI signaling pathway, and B cell receptor signaling pathway, as well as immune responses(**Figure 3e**). In the PPI network (**Figure 3f**), the hub proteins with the highest connectivity included FXR1, EIF3B, ANPEP, and CD9. Among them, FXR1 is an RNA-binding protein involved in post-transcriptional regulation, while EIF3B constitutes a component of the eukaryotic translation initiation factor eIF3 complex and participates in translational control. ANPEP functions in peptide degradation and signal transduction, and CD9 acts as a co-receptor in B lymphocytes, contributing to immune regulation. Integrated with KEGG findings, these proteins may collectively contribute to phenotypic differences between PTC with HT and HT by engaging in regulatory interactions that are not directly associated with canonical signaling pathways.

**Figure 4:**
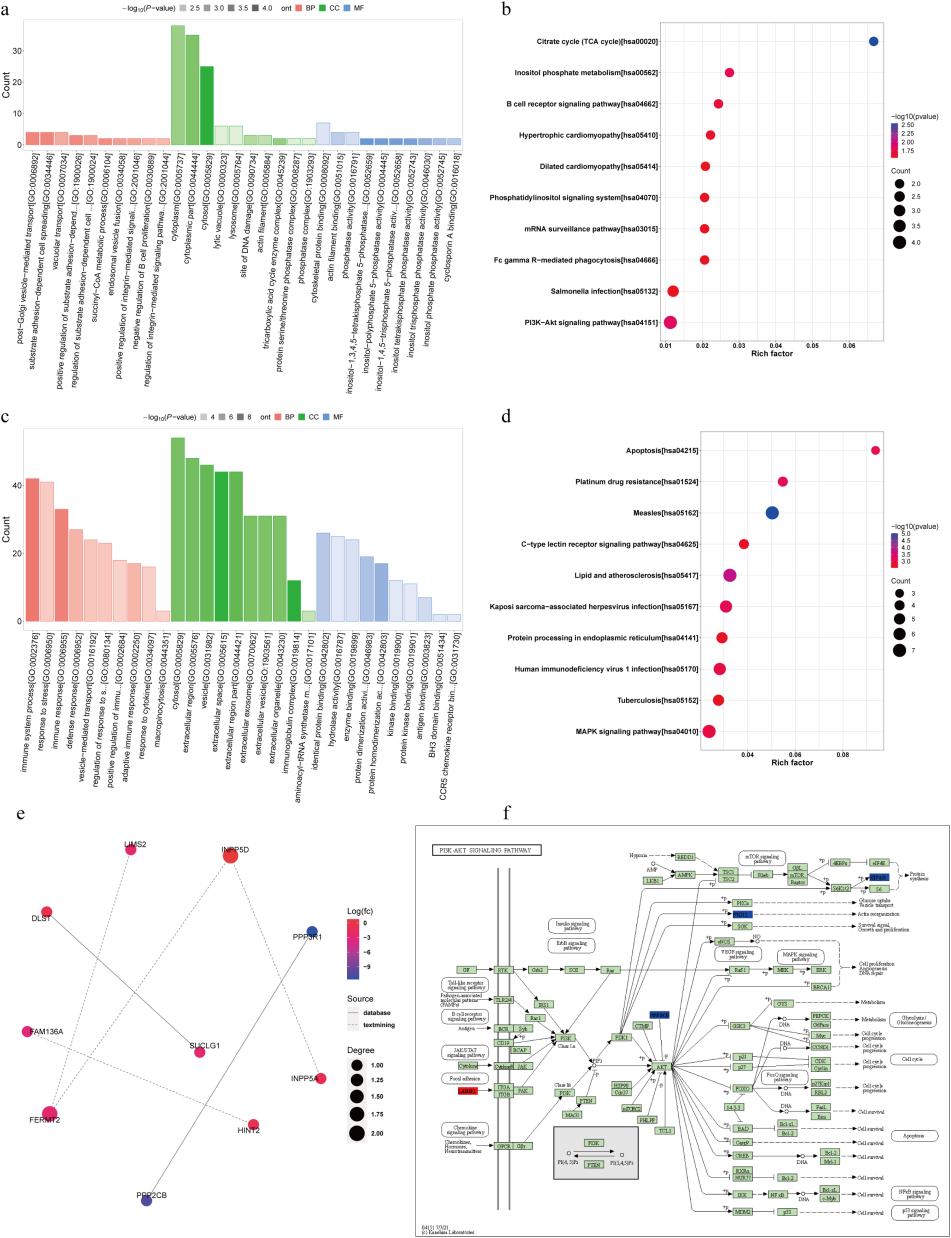
Functional enrichment and protein-protein interaction analysis of DEPs. **(a)** GO term enrichment analysis of DEPs identified in the PTC vs PTC-W comparison. **(b)** KEGG pathway enrichment analysis of DEPs identified in the PTC vs PTC-W comparison. **(c)** GO term enrichment analysis of DEPs identified in the PTC vs HT comparison. **(d)** KEGG pathway enrichment analysis of DEPs identified in the PTC vs HT comparison. **(e)** Protein-protein interaction (PPI) network of DEPs identified in the PTC vs PTC-W comparison, constructed using the STRING database. **(f)** Schematic diagram of the PI3K-AKT signaling pathway, retrieved from the KEGG PATHWAY database (https://www.kegg.jp/kegg/pathway.html; entry: hsa04151).

Comparative analysis of DEPs between PTC and PTC-W as well as between PTC and HT, revealed the following findings. In the PTC vs PTC-W comparison, 3 proteins were upregulated and 41 proteins were downregulated in PTC relative to PTC-W. In the PTC vs HT comparison, 34 proteins were upregulated and 298 proteins were downregulated in PTC relative to HT. The GO functional analysis indicated that the biological processes associated with the DEPs in PTC-W (**Figure 4a**) were predominantly related to substance transport, while the enriched cellular components were mainly localized in the cytoplasm. In contrast, DEPs associated with HT (**Figure 4c**) were primarily involved in immune-related processes, and major cellular components were distributed in the cytoplasm and extracellular regions. In the MF category, DEPs in PTC-W were enriched in cytoskeletal protein binding, actin filament binding, and phosphatase activity, which are closely associated with extracellular matrix organization and signal transduction. Meanwhile, DEPs in HT were mainly enriched in identical protein binding, hydrolase activity, enzyme binding, and protein dimerization activity, which are highly involved in signaling-related molecular interactions. The KEGG pathway enrichment analysis further revealed that DEPs in PTC-W (**Figure 4b**) were predominantly associated with the PI3K–Akt signaling pathway and inositol phosphate metabolism. In contrast, DEPs in HT (**Figure 4d**) were mainly enriched in the MAPK signaling pathway, protein processing in the endoplasmic reticulum, and the C-type lectin receptor signaling pathway. The PPI network analysis of DEPs between PTC and PTC-W (**Figure 4e**) indicated that FERMT2 and INPP5D functioned as central hub genes in the network. Notably, INPP5D, an inositol polyphosphate-5-phosphatase, could directly inhibit the PI3K-Akt signaling pathway by hydrolyzing PIP3 (**Figure 4f**). Moreover, INPP5A was found to interact with INPP5D, collectively influencing proteomic expression.

**Figure 5:**
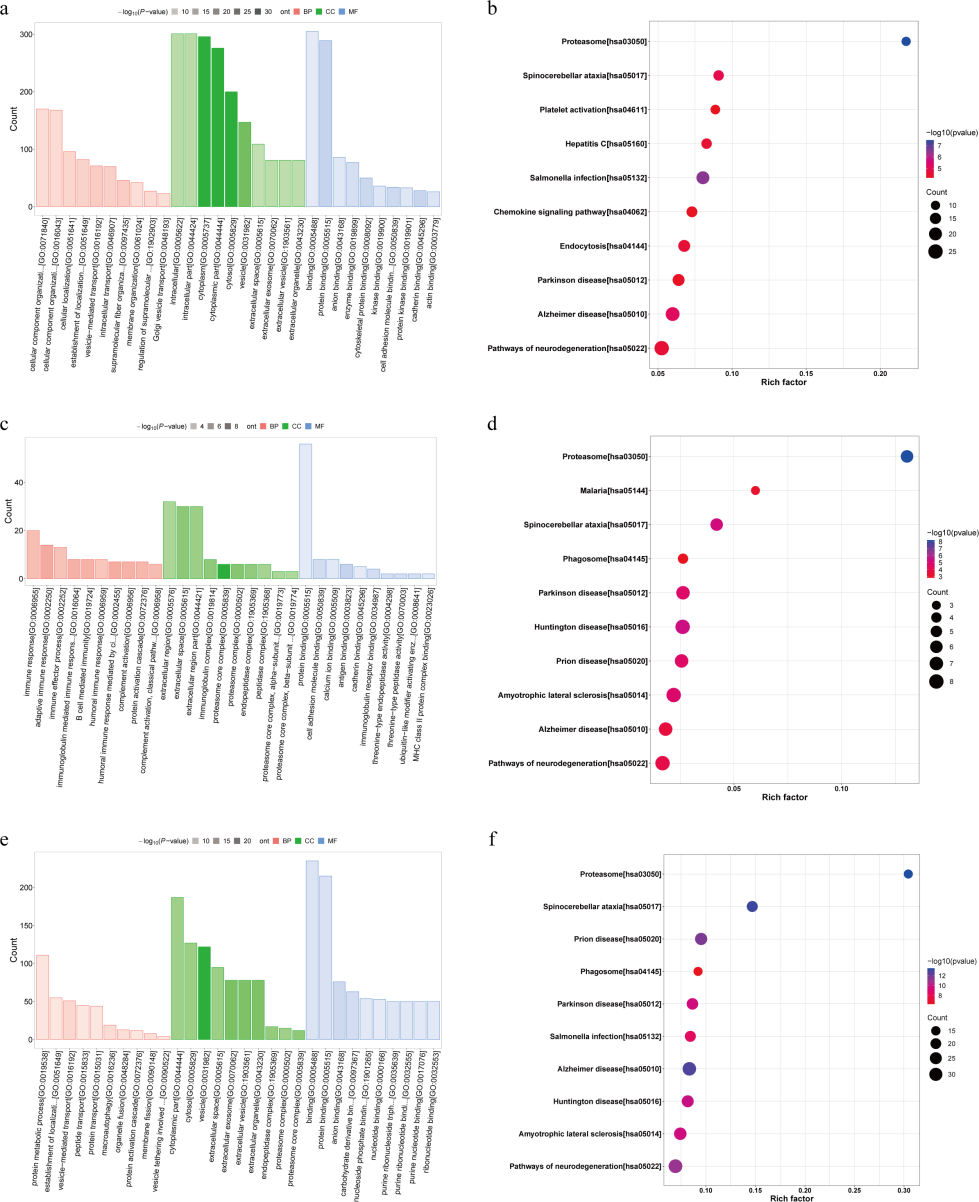
GO term enrichment analysis of DEPs identified in **(a)** PTC vs HC, **(c)** PTC-W vs HC, and **(e)** HT vs HC comparisons. KEGG pathway enrichment analysis of DEPs identified in **(b)** PTC vs HC, **(d)** PTC-W vs HC, and **(f)** HT vs HC comparisons.

**Figure 6:**
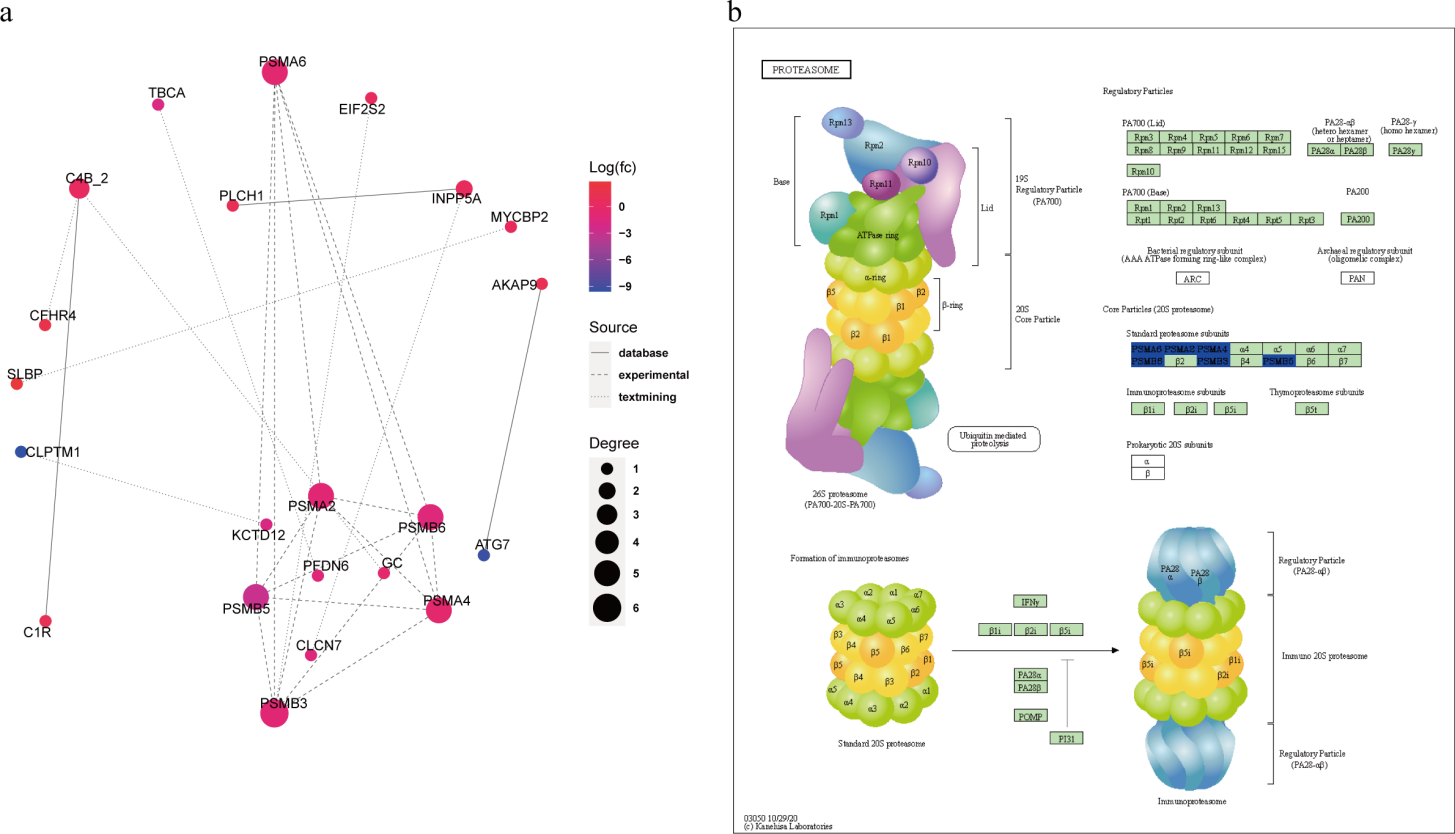
The DEPs between PTC-W and HC. **(a)** The interacted network of proteins analyzed by STRING. **(b)** The composition and structure of the proteasome.This figure is a manually curated pathway mapretrieved from the KEGG PATHWAY database (https://www.kegg.jp/kegg/pathway.html entry: hsa03050).

Compared with HC, the DEPs in PTC, PTC-W, and HT were analyzed. The GO analysis revealed that in BPs, DEPs in PTC (**Figure 5a**) were primarily involved in cellular component organization and biogenesis, while DEPs in HT (**Figure 5e**) were associated with protein metabolism, cellular localization, and vesicle-mediated transport. The primary cellular components of the DEPs in both PTC and HT were localized intracellularly. In contrast, DEPs in PTC-W (**Figure 5b**) were predominantly involved in immune responses and complement activation, in which the main cellular components were found in the extracellular region. Regarding MFs, DEPs in all the three groups were involved in cell and protein adhesion. Additionally, PTC-W participated in calcium ion metabolism. The KEGG pathway analysis revealed that the DEPs in PTC-W and HT were mainly enriched in the proteasome and phagosome signaling pathways, while PTC was also involved in the chemokine signaling pathway and endocytosis (**Figures 5b, d, f**). PPI analysis of DEPs between PTC-W and HC (**Figure 6a**) indicated that PSMB3, PSMB5, PSMA2, PSMA4, PSMA6, and PSMB6 functioned as core proteins. These proteins participated in proteasome assembly (**Figure 6b**), primarily promoting protein degradation, regulating cell cycle progression, and controlling cellular growth.

### 3.4 Verification of plasma protein signatures in thyroid disease

**Figure 7:**
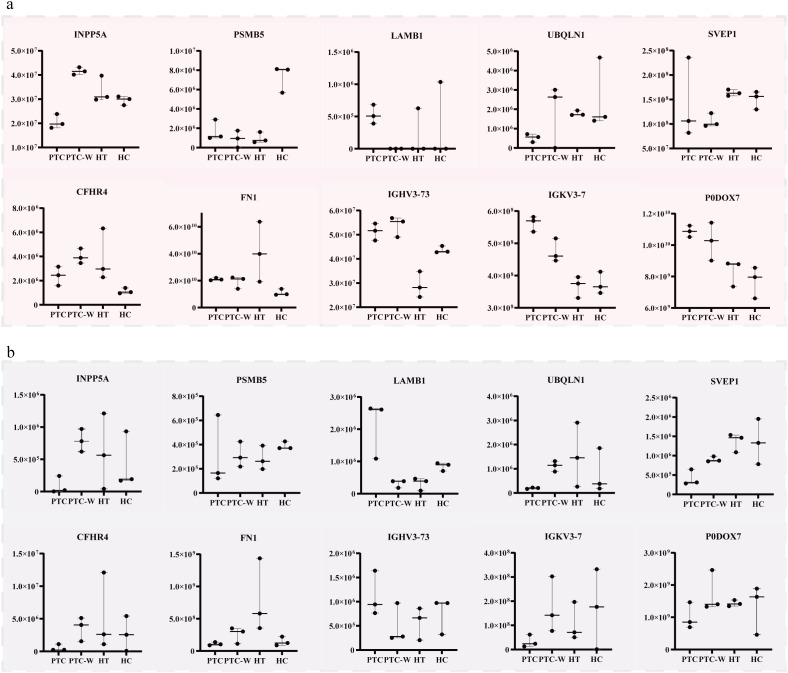
Scatter plots of the ten validated proteins from **(a)** label-free and **(b)** PRM quantification. Each panel shows individual data points (n = 3 per group), medians (horizontal lines), and interquartile ranges (error bars) for the four groups (PTC, PTC-W, HT, HC). For measurements below the detection limit, platform-specific constant imputed values were used. Medians and IQRs were calculated from all available data, including imputed constants.

To select candidates for PRM validation, we performed label-free proteomic comparisons across multiple groups. Candidate proteins were prioritized based on statistical significance (P < 0.05), large fold changes, and distinctive expression patterns across multiple groups. This multi-step filtering process yielded 20 candidates for PRM validation, including SVEP1 and IGKV3-7. The PRM validation was performed on the same patient cohort as the label-free discovery set. To validate the reliability of the label-free quantitative analysis, PRM analysis was conducted on these 20 candidates. The complete list is as follows: IGKV3-7, RRAGB, INPP5A, PSMB5, AP1G1, C2CD5, LAMB1, ATAD2, UBQLN1, IGHV3-15, SVEP1, CFHR4, FN1, HLA-DPA1, IGHV3-49, IGHV3-73, MAP1S, P0DOX7 (UniProt ID), and SUN3. Among the validated proteins, INPP5A, PSMB5, LAMB1, UBQLN1, SVEP1, CFHR4, FN1, IGHV3-73, IGKV3-7, and P0DOX7 exhibited consistent directional changes between PRM and label-free quantification (**Table 2**), and the results were consistent with those obtained from the label-free quantitative analysis. The distribution of individual sample values, medians, and interquartile ranges for these ten validated proteins is shown in scatter plots (**Figure 7**).

**Table 2:** PRM validation results of candidate proteins and comparison with label-free data.

Gene name	Accessions	Comparison	FC (PRM)	P value (PRM)	FC (lable-Free)	P value (Lable-Free)	Trend consistency	Significance	Regulation
INPP5A	Q14642	B_vs_D	2.18	0.144	1.41	0.001	Consistent	Significant	↑
INPP5A	Q14642	A_vs_B	0.11	0.005	0.49	0.000	Consistent	Significant	↓
PSMB5	P28074	B_vs_D	0.51	0.248	0.12	0.003	Consistent	Significant	↓
LAMB1	P07942	A_vs_B	6.65	0.071	474.46	0.025	Consistent	Significant	↑
UBQLN1	Q9UMX0	A_vs_C	0.13	0.221	0.29	0.001	Consistent	Significant	↓
SVEP1	Q4LDE5	B_vs_C	0.40	0.027	0.65	0.003	Consistent	Significant	↓
CFHR4	Q92496	B_vs_D	1.33	0.658	3.53	0.002	Consistent	Significant	↑
CFHR4	Q92496	A_vs_B	0.15	0.050	0.60	0.049	Consistent	Significant	↓
FN1	P02751	A_vs_D	1.24	0.255	1.90	0.003	Consistent	Significant	↑
IGHV3-73	A0A0B4J1V6	C_vs_D	0.76	0.572	0.67	0.010	Consistent	Significant	↓
IGHV3-73	A0A0B4J1V6	A_vs_C	1.94	0.178	1.76	0.004	Consistent	Significant	↑
IGKV3-7	A0A075B6H7	B_vs_D	1.02	0.976	1.27	0.025	Consistent	Significant	↑
IGKV3-7	A0A075B6H7	B_vs_C	1.63	0.452	1.29	0.020	Consistent	Significant	↑
	P0DOX7	B_vs_D	1.31	0.518	1.33	0.049	Consistent	Significant	↑

Label-free DEPs: unique peptide ≥ 1, fold change ≥ 1.2 or ≤ 0.83, P < 0.05. PRM: fold change ≥ 1 or ≤ 1 (no strict cutoff due to small sample size; validation focused on trend consistency). Consistent” indicates that the direction of regulation (up-regulated or down-regulated) for a given protein is the same between the label-free and PRM measurements. “FC” stands for fold change. A, PTC; B, PTC-W; C, HT; D, HC. ↑, upregulation (higher expression in the firstnamed group relative to the second); ↓, downregulation (lower expression).

## 4 Discussion

PTC is the most common thyroid malignant tumor, while HT represents the most prevalent autoimmune disease in humans. Previous research has demonstrated that the co-occurrence rate of PTC with HT averages approximately 23% (range, 5-85%) (11). It is therefore necessary to identify diagnostic biomarkers to recognize high-risk populations developing PTC among HT groups.

In our study, proteomic analysis was performed on plasma samples from patients with PTC, PTC-W, HT, and HC. A total of 2,750 proteins were analyzed and quantified, revealing significant differences in protein expression among the groups. Functional annotation and enrichment analysis of the PRM-validated DEPs (listed in **Table 2**) indicated that these proteins are primarily involved in extracellular matrix-receptor interactions, immune responses, and proteasome activities. Further validation highlighted SVEP1 and IGKV3–7 as proteins potentially implicated in the transition from HT to PTC.

In the present study, the expression level of SVEP1 was decreased in PTC-W group compared with HT group. SVEP1 is located in the 9q32 region of chromosome 9 and encodes the extracellular matrix protein POLYDOM. As a multi-domain adhesion molecule in the extracellular matrix, SVEP1 mediates cell-cell and cell-matrix interactions via its von Willebrand factor type A (vWA) and epidermal growth factor (EGF)-like domains (12). It is involved in vascular physiology and lymphatic vessel development. Recent studies have further revealed that SVEP1 binds to integrins α4β1 and α9β1, thereby reducing vascular smooth muscle cell contractility by suppressing PRKCA-mediated calcium influx through L-type voltage-gated calcium channels (VGCCs) and by reducing ROCK-mediated calcium sensitivity (13). In addition, SVEP1 can upregulate the expression levels of Hey2 and Hes1 in the Notch signaling pathway, promoting vascular smooth muscle cell proliferation (14). SVEP1 directly binds to Tie1 in the angiopoietin–tie axis and promotes lymphatic endothelial cell (LEC) migration in a Tie1-dependent manner (15). Under inflammatory conditions, SVEP1 directly interacts with Angpt1, attenuating Angpt1-mediated leukocyte adhesion and thereby reducing leukocyte recruitment (16). SVEP1 deficiency also upregulates endothelial CXCL1 expression, further promoting chemotaxis of inflammatory leukocytes to the diseased area (17). In tumor cells, low expression level of SVEP1 can promote the migration, chemotaxis, invasion, and proliferation of cancer cells *in vitro*. A prior study has demonstrated that the decreased expression level of SVEP1 could enhance Akt phosphorylation at Thr308, thereby activating the PI3K/Akt signaling pathway and inducing proliferation and metastasis of hepatocellular carcinoma (HCC) cells. Additionally, SVEP1 expression level can be regulated by mir-1269b (18). In colorectal cancer, lncRNA BVES-AS1 functions as a ceRNA to sequester mir-1296b, thereby competitively regulating SVEP1 (19). Beyond directly affecting cancer cell behavior, SVEP1 may also reshape the tumor microenvironment by regulating immune cell differentiation. It has been shown that SVEP1 expression is significantly upregulated during monocyte-to-macrophage differentiation, and it regulates monocyte adhesion, migration, and differentiation phenotype via integrin α4β1/α9β1 and Rho/Rac signaling (20). The decreased expression level of SVEP1 in PTC-W compared with HT was found to be consistent with previously reported findings, promoting cancer cell proliferation. Considering the regulatory roles of SVEP1 in leukocyte recruitment and monocyte differentiation under inflammatory conditions, we hypothesize that downregulation of SVEP1 in the chronic inflammatory microenvironment of Hashimoto’s thyroiditis may alter the polarization and function of tumor-associated macrophages (TAMs), thereby indirectly promoting PTC progression. This proposed mechanism warrants further investigation.

The present study revealed that plasma level of IGKV3–7 was significantly elevated in patients with PTC-W compared with those with HT. Importantly, a search of the Vesiclepedia database (http://www.microvesicles.org) confirms that IGKV3-7 has previously been identified as a protein cargo of human plasma-derived small extracellular vesicles using mass spectrometry and ultracentrifugation (21), which is consistent with our own isolation and detection methods. IGKV3–7 is a subfamily of immunoglobulin kappa light chain genes (IGKV) that participates in antigen recognition by the variable region V of immunoglobulin light chains. The variable domains of immunoglobulins are assembled through a process called V-J recombination and can subsequently undergo somatic hypermutation. Following antigen exposure and selection, this enables affinity maturation against specific antigens. The expression level of IGKV3–7 may be associated with the pathogenesis of ankylosing spondylitis (22). Evidence demonstrated that IGKV3–7 could play a role in the pathophysiological process of osteoporosis by participating in immune system regulation (23).This elevated plasma level may be associated with immune responses triggered by tumor cells, indicating the potential of IGKV3–7 as an early diagnostic biomarker for PTC complicated with HT. Notably, no significant correlation was observed between IGKV3-7 expression and serum TPOAb/TgAb titers. This finding suggests that exosomal IGKV3-7 may reflect local immune status within the thyroid microenvironment rather than systemic autoantibody levels. However, owing to the limited sample size, these results require validation in larger independent cohorts.

In this study, the identified DEPs in the extracellular matrix also included LAMB1 and FN1. FN1 was found to be significantly upregulated in the PTC group compared with the HC group. FN1 encodes fibronectin in the extracellular matrix. The RGD domain of FN1 binds to integrin α5β1, activating Ras protein and subsequently triggering the MAPK and PI3K-Akt-mTOR signaling pathways (24). It can also promote tumor cell generation by activating the NF-κB signaling pathway (25). Additionally, FN1 acts on vascular endothelial cells (26) to facilitate tumor metastasis. However, previous research indicated that FN1 binding to integrin α5β1 could activate the IKKα/β-JNK1-BECN1 signaling pathway, thereby mediating hepatic autophagy (27) and suppressing tumor growth. The EDB domain of FN1 is regulated by the *ZMAT3* gene, upregulating p21 expression level to inhibit the growth of PTC cells (28). These opposing roles of FN1 in tumor biology suggest context-dependent effects. In the present study, LAMB1 expression was upregulated in the PTC group compared with the PTC-W group. LAMB1 encodes laminin β in the extracellular matrix, serving as a major component of the extracellular matrix. LAMB1 can activate the PI3K-AKT signaling pathway through the focal adhesion signaling pathway, thereby regulating cell growth, metabolism, and apoptosis. The expression level of LAMB1 is upregulated in various cancer types, which may be attributed to the IRES sequence in the UTR region of LamB1, enhancing its transcription level during tumor progression (29). Scholars have shown that RNA helicase DDX24 binds to LAMB1 mRNA and enhances its stability, thereby promoting tumor cell development (30). Additionally, LAMB1 mediates endothelial cell proliferation via the Wnt/β-catenin signaling pathway, promoting the formation of new blood vessels in tumors (31). Notably, the upregulation of LAMB1 in PTC relative to PTC-W in the study might be associated with extensive cell destruction in HT. A study revealed that laminin staining surrounding damaged thyroid follicles in HT patients was significantly reduced and exhibited a negative correlation with the extent of lymphocyte infiltration (32).

The immune system comprises the innate immune system and the adaptive immune system, and the complement system is a component of the innate immune system. In PTC, the elevated expression levels of C3, C4, and C5b-9 have been found, and excessive activation of the complement lectin pathway and the alternative pathway could contribute to the pathogenesis of the disease (33). In the present study, CFHR4 expression level was elevated in PTC patients with HT compared with HCs and patients with only PTC. CFHR4 competes with complement factor H (CFH) for binding to C3b, resulting in overactivation of the complement system. Numerous studies have demonstrated that CFHR4 plays a role in immune system-associated diseases, such as age-related macular degeneration, systemic lupus erythematosus, and atypical hemolytic uremic syndrome. Research has also shown that CFHR4 expression level was reduced in HCC cells and was significantly associated with poor prognosis and immune infiltration level in HCC (34). Regarding adaptive immunity, IGHV3-73 expression was low in the HT group but significantly upregulated in the PTC group compared with HT. IGHV3-73, which is associated with adaptive immunity, is a subfamily of immunoglobulin heavy chain variable region genes (IGHV) involved in antigen recognition of the variable region V of immunoglobulin heavy chains. IGHV3–73 is commonly used in the diagnosis of chronic lymphocytic leukemia (35). Studies have shown that IGHV3–73 expression level was upregulated in B lymphocytes of patients with malaria (36), while it was downregulated in patients with luminal A breast cancer (37). P0DOX7, which encodes an immunoglobulin κ light chain, was found to be elevated in the PTC-W group compared with the HC group. High expression of P0DOX7 may be associated with poor prognosis in multiple sclerosis (38).

The ubiquitin-proteasome system is involved in the degradation of misfolded proteins in thyroid diseases, regulating the cell cycle and controlling cell growth.

In the present study, PSMB5 was downregulated in PTC-W compared with HT. PSMB5 encodes the proteasome β5 subunit, and previous research has shown that PSMB5 expression level was upregulated in HCC, prostate cancer, and breast cancer cells. In inflammatory diseases, such as autoimmune thyroid diseases, the elevated IL-17 level activates the NF-κB signaling pathway, stimulating the production and release of inflammatory factors (e.g., IFN-γ) (39). When cells are stimulated by inflammatory factors, such as IFN-γ, the β5i subunit encoded by PSMB8 replaces the β5 subunit, forming the immunoproteasome (40), andPSMB5 expression level is downregulated. PSMB8 participates in MHC class I antigen presentation, promotes T cell maturation and differentiation, engages in inflammatory responses, and influences tumor cell growth (41). Scholars have pointed out that downregulation of PSMB8 expression level could inhibit the growth, epithelial-mesenchymal transition, and induction of apoptosis in PTC (42).

The downregulation of PSMB5 in PTC-W patients in the study could be associated with the formation of immunoproteasomes in the context of HT. In the present study, UBQLN1 was found to be elevated in the HT group compared with the PTC group. UBQLN1 recognizes ubiquitinated proteins in the endoplasmic reticulum and promotes their delivery to the proteasome. UBQLN1 induces the degradation of PGC1β, thereby suppressing mitochondrial biogenesis and the generation of reactive oxygen species (ROS), ultimately mediating cell apoptosis (43). Silencing UBQLN1 reduces the expression level of the P53 protein, subsequently promoting cell proliferation (44). In HT, NF-κB signaling pathway is activated (39), and the elevated UBQLN1 expression level can regulate NF-κB signaling pathway by promoting the proteasomal degradation of its RelA subunit via the immunoproteasome (45). The upregulation of UBQLN1 in HT group in the study compared with PTC group may reflect its role in modulating the activity of NF-κB signaling pathway.

In this study, the expression level of INPP5A was upregulated in PTC-W group compared with PTC group and HT group. INPP5A regulates intracellular calcium ion metabolism through PIP2 hydrolysis, thereby maintaining cellular homeostasis. The deficiency of INPP5A leads to IP3 accumulation, resulting in hyperactivation of IP3/Ca2+ signaling and promotion of p53-dependent apoptotic cell death (46).This upregulation of INPP5A in PTC-W could be related to the increased incidence of PTC in HT as reported by Roberto (47). The outcomes provide a potential basis for analyzing the mechanism by which HT promotes PTC.

The present study identified SVEP1 and IGKV3–73 as potential biomarkers for diagnosing PTC in the context of HT by analyzing DEPs between PTC-W and HT. In contrast, Li et al. (48) reported MET and FAM20A as common DEPs among PTC, HT, and PTC-W. For experimental validation, they employed immunohistochemistry (IHC), whereas the current investigation utilized targeted proteomics via PRM. Compared with IHC, PRM enables the absolute quantification of target proteins and allows for precise detection of subtle differences in protein expression. Nevertheless, this study has certain limitations, including a single-center design and a limited sample size, and the fact that the PRM validation was conducted in the same cohort without an independent external validation(EV) set. In addition, as acknowledged in the MISEV2023 guidelines, exosomes were isolated by ultracentrifugation, which is a relatively crude method that may co-pellet non-EV contaminants such as abundant plasma proteins and lipoproteins. We did not systematically assess these contaminants (e.g., albumin, apolipoproteins), which constitutes a methodological limitation. Therefore, the proteomic data may include signals from non-EV components, and further validation using more stringent purification methods (e.g., density gradient ultracentrifugation or size-exclusion chromatography) is warranted. Collectively, these limitations indicate the necessity of further validation in larger, independent cohorts and with improved EV purification protocols.

## 5 Conclusion

This study carried out an integrated proteomics analysis of plasma exosomes to systematically compare PTC-W and HT. A total of 45 DEPs were successfully identified. Subsequently, bioinformatics analysis was conducted. KOG analysis demonstrated that the DEPs primarily participated in posttranslational modification and protein turnover. GO analysis revealed that they were significantly enriched in responses to stimulus and functions associated with immunoglobulin complexes. Further KEGG pathway analysis disclosed significant clustering of the mTOR signaling pathway, Fc epsilon RI signaling pathway, and BCR signaling pathway. Moreover, through PRM-targeted proteomics validation, SVEP1 and IGKV3–7 were identified as two key proteins with significant differential expression between groups. Specifically, in the key comparison between PTC-W and HT, SVEP1 was downregulated and IGKV3-7 was upregulated in PTC-W relative to HT.Among them, the downregulation of SVEP1 was associated with tumor cell growth, suggesting that it plays a vital role in cell adhesion and microenvironment regulation. Meanwhile, as a component of the immunoglobulin light chain, the alteration of IGKV3–7 provided direct molecular clues for the aforementioned dysregulated BCR signaling pathway, highlighting the potential role of B cell activity changes in disease progression. This study suggests that an imbalance in protein homeostasis and a disorder of the immune microenvironment may be implicated in the development of PTC in the context of HT. Furthermore, this study is the first to identify plasma exosome-derived SVEP1 and IGKV3–7 as potential specific biomarkers for distinguishing between these two diseases. As candidate biomarkers, SVEP1 and IGKV3–7 provide a rationale for developing simpler, high-throughput detection methods. In the future, these two protein markers should be validated in larger, independent cohorts using cost-effective and readily automatable targeted immunoassays. Moreover, integrating these biomarkers with routine clinical parameters (e.g., ultrasound findings and serum autoantibody levels) may improve diagnostic accuracy for distinguishing PTC-W from HT. In addition, in-depth research on the specific functional mechanisms of SVEP1 and IGKV3–7 within the microenvironment of thyroid tumors is crucial for understanding the transformation process from HT to PTC.

## Data Availability

The original contributions presented in the study are included in the article/**Supplementary Material**. Further inquiries can be directed to the corresponding author/s.
