# Supplementary figures and images for: Integrative discovery and targeted proteomics elucidate the plasma exosomal landscape in thyroid disorders, with emphasis on papillary thyroid carcinoma complicating Hashimoto’s thyroiditis
Source: Front Endocrinol (Lausanne). 2026 May 25;17:1778949. doi: 10.3389/fendo.2026.1778949 (PMC13243065; doi:10.3389/fendo.2026.1778949)

RT: 0.00 - 60.01

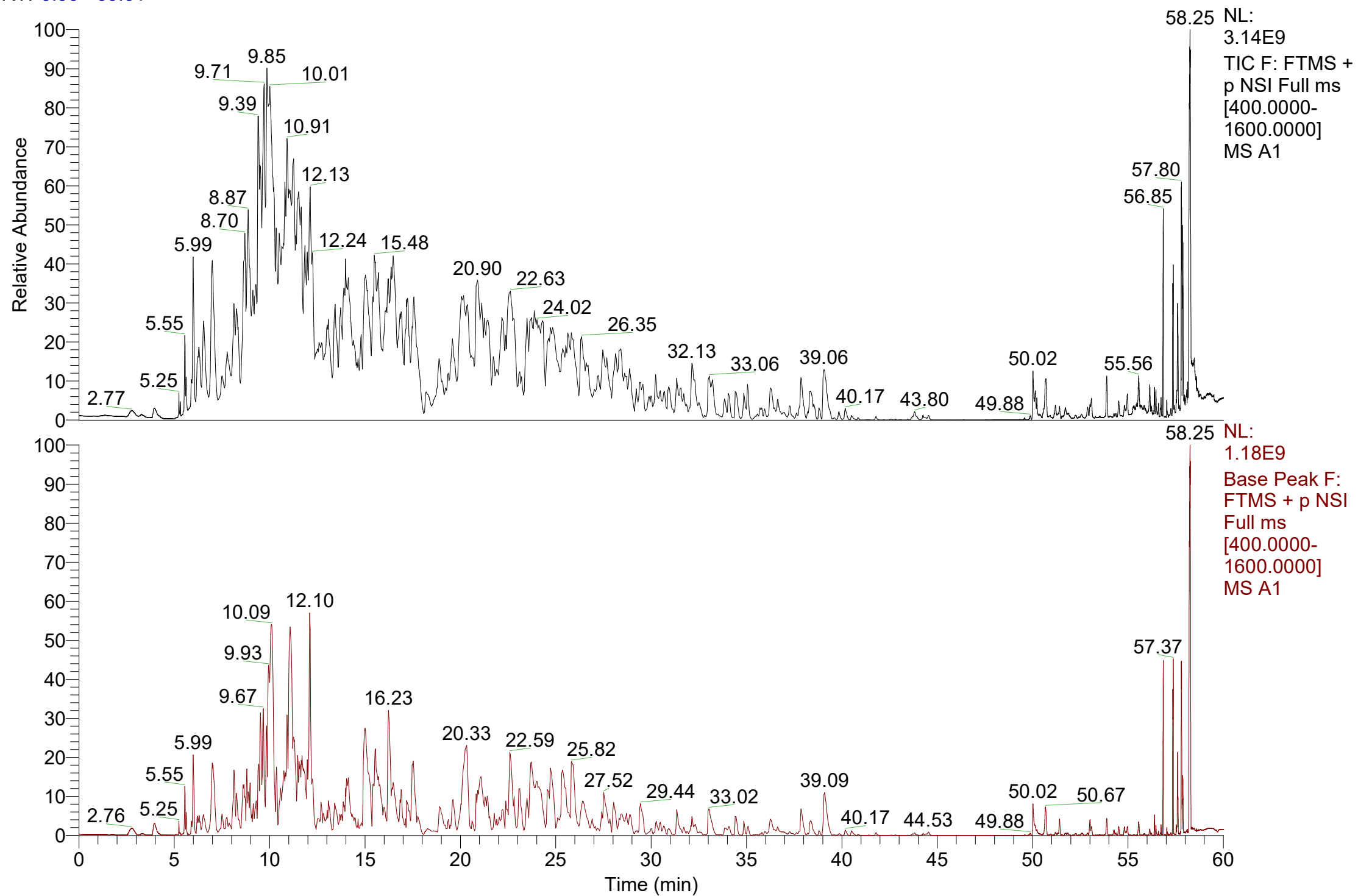

Supplement: Supplementary file 1 [file DataSheet1.pdf]

RT: 0.00 - 60.00

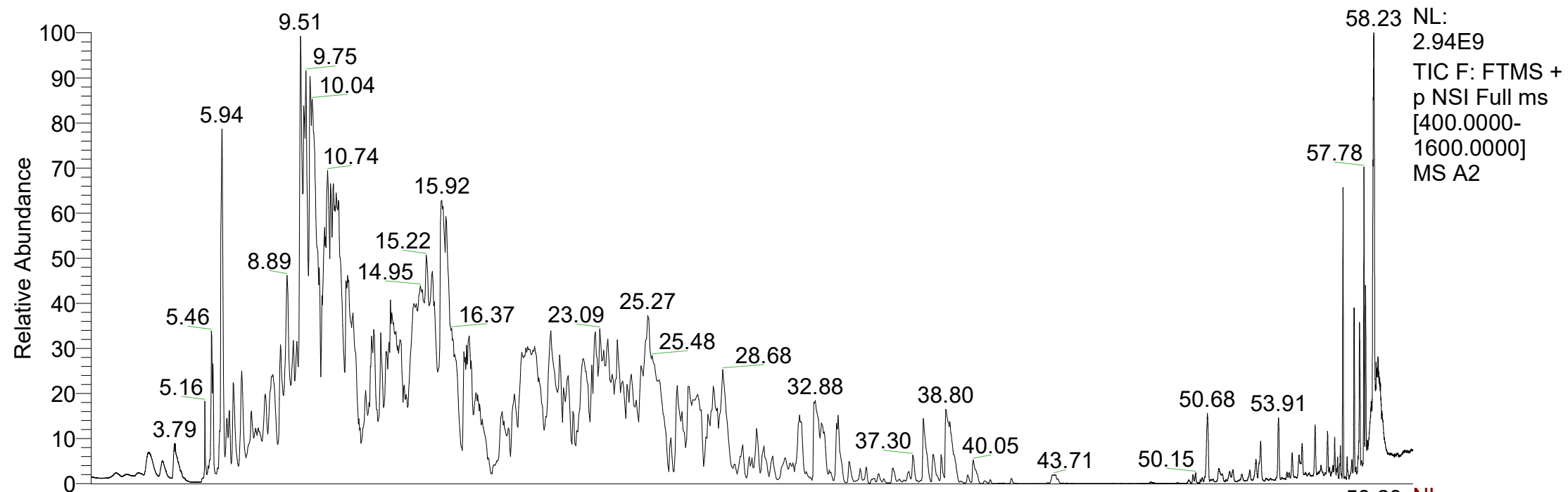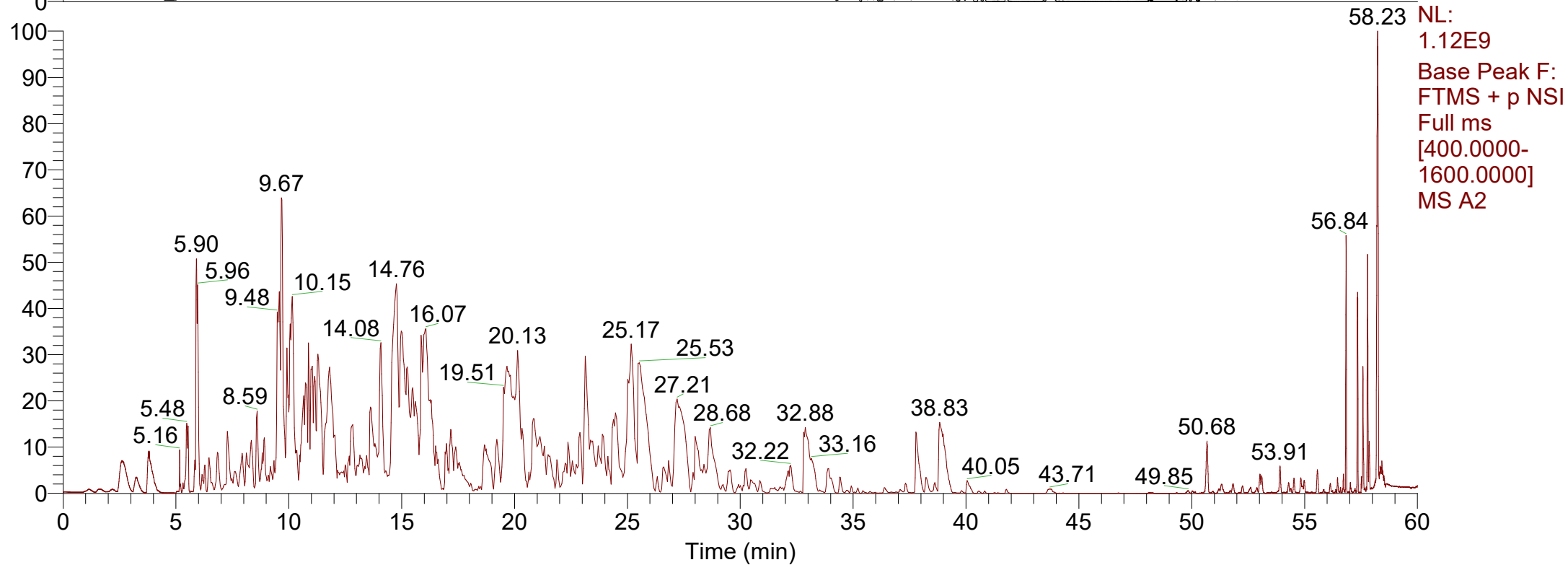

Supplement: Supplementary file 2 [file DataSheet2.pdf]

RT: 0.00 - 60.00

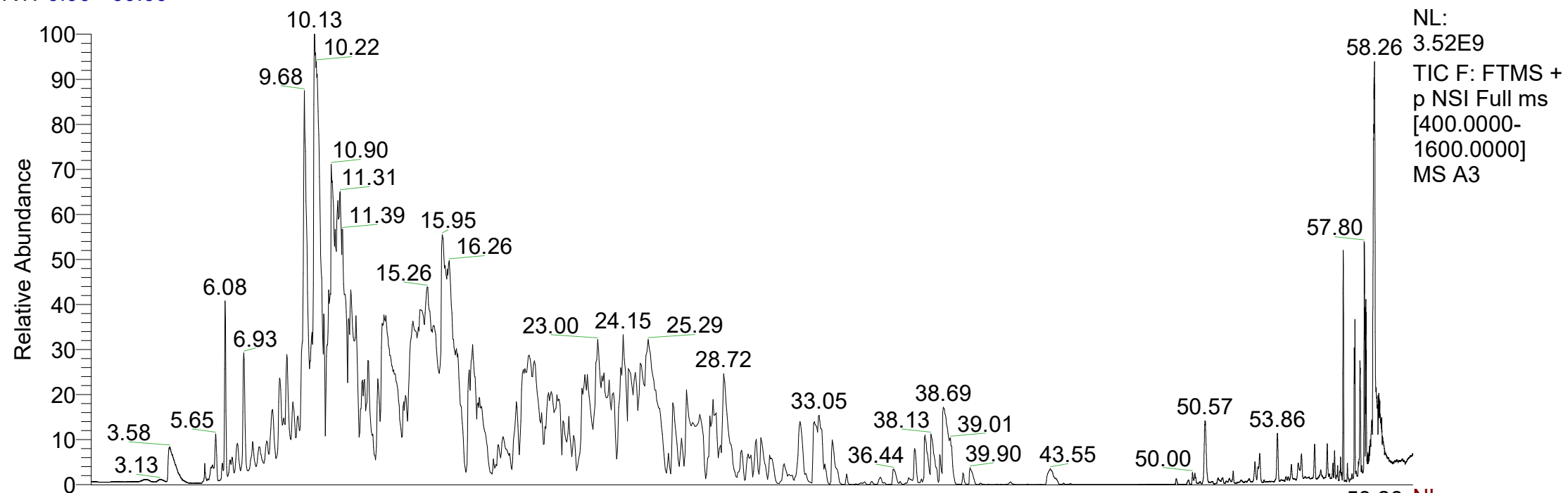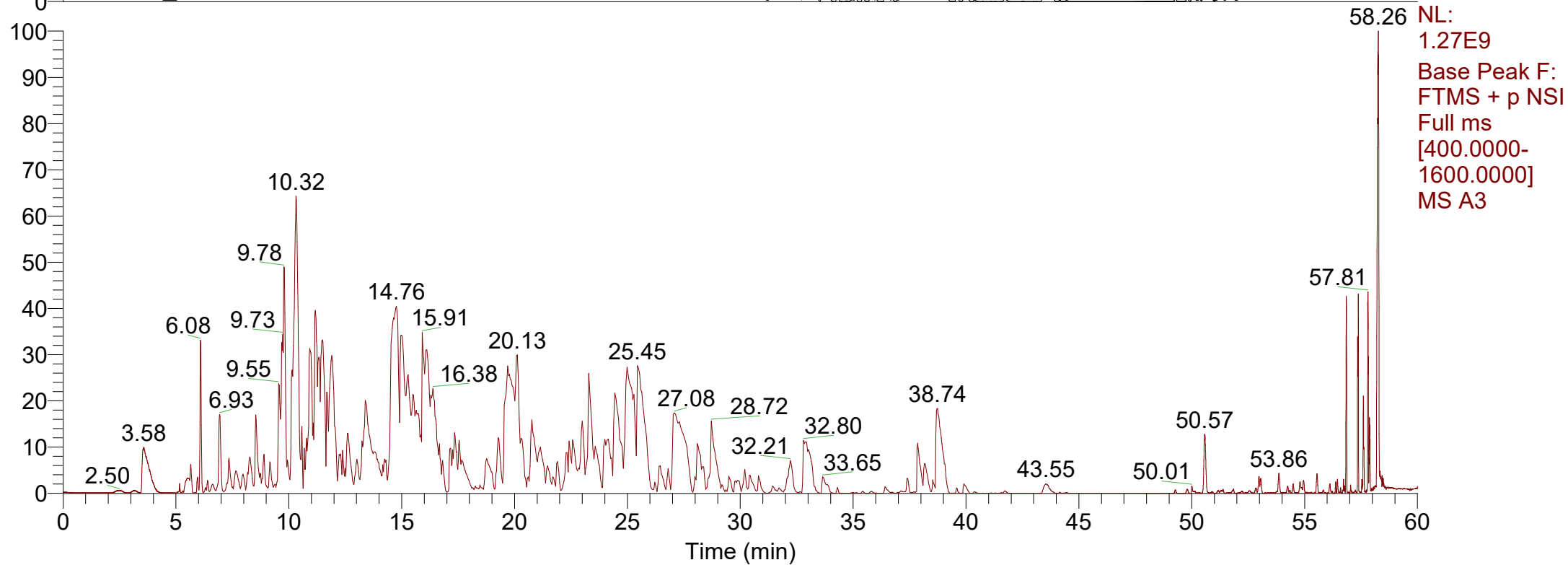

Supplement: Supplementary file 3 [file DataSheet3.pdf]

RT: 0.00 - 60.00

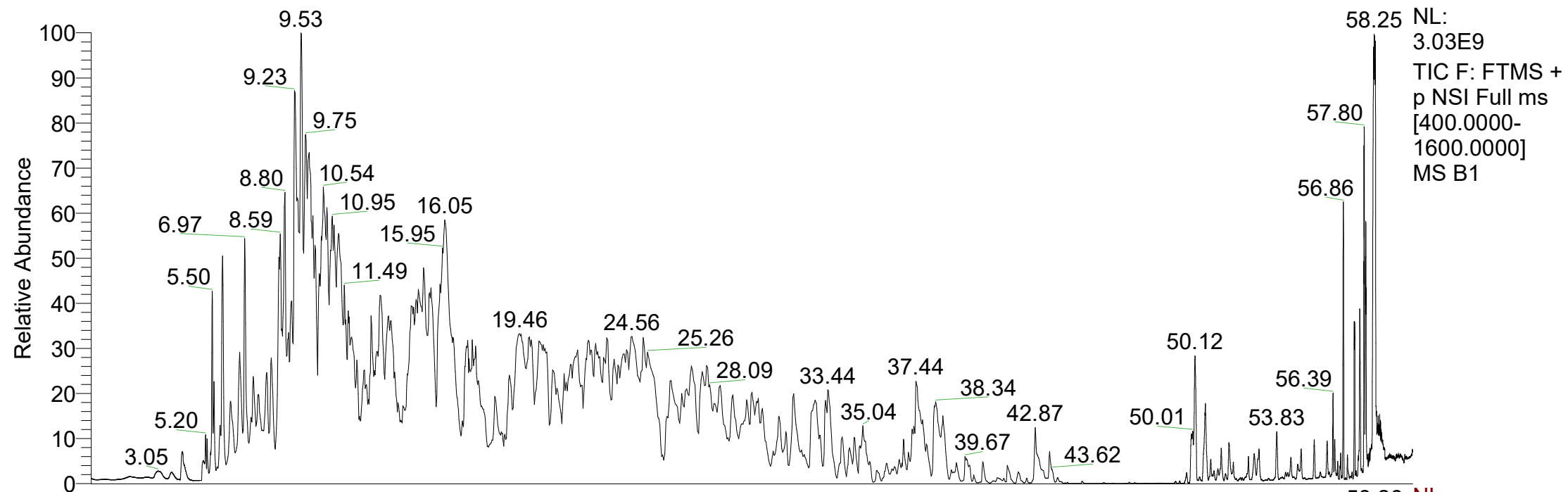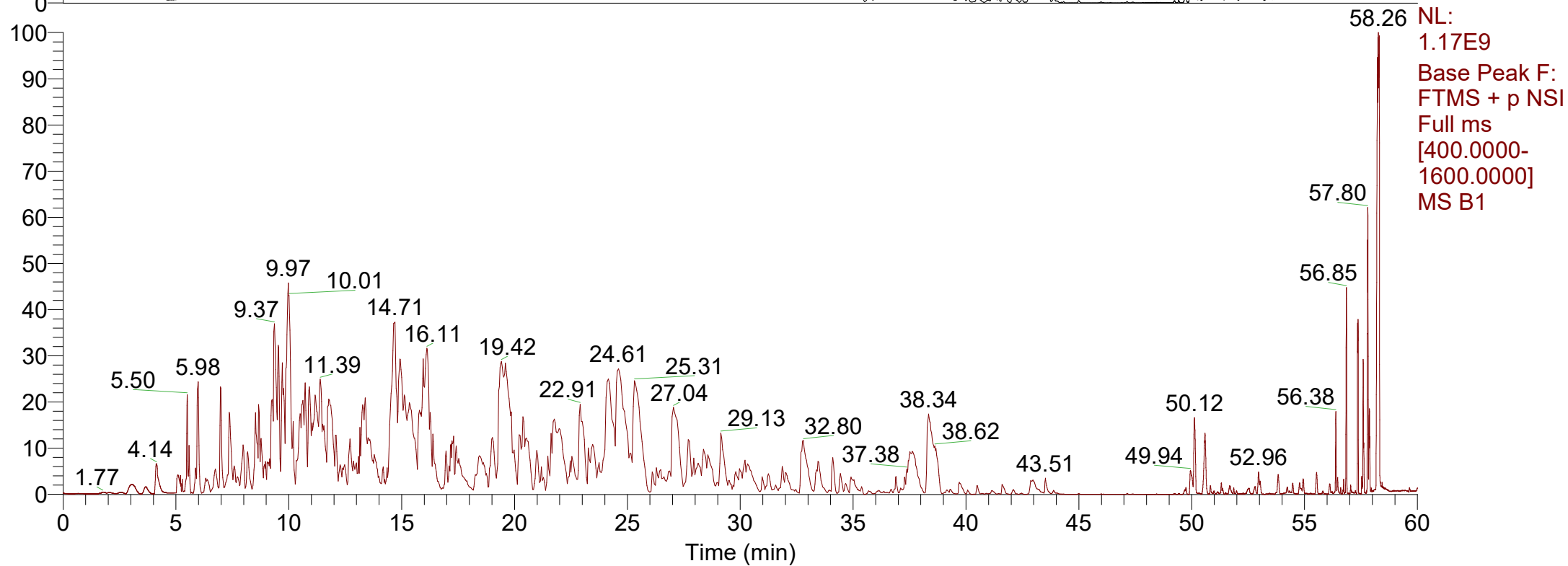

Supplement: Supplementary file 4 [file DataSheet4.pdf]

RT: 0.00 - 60.00

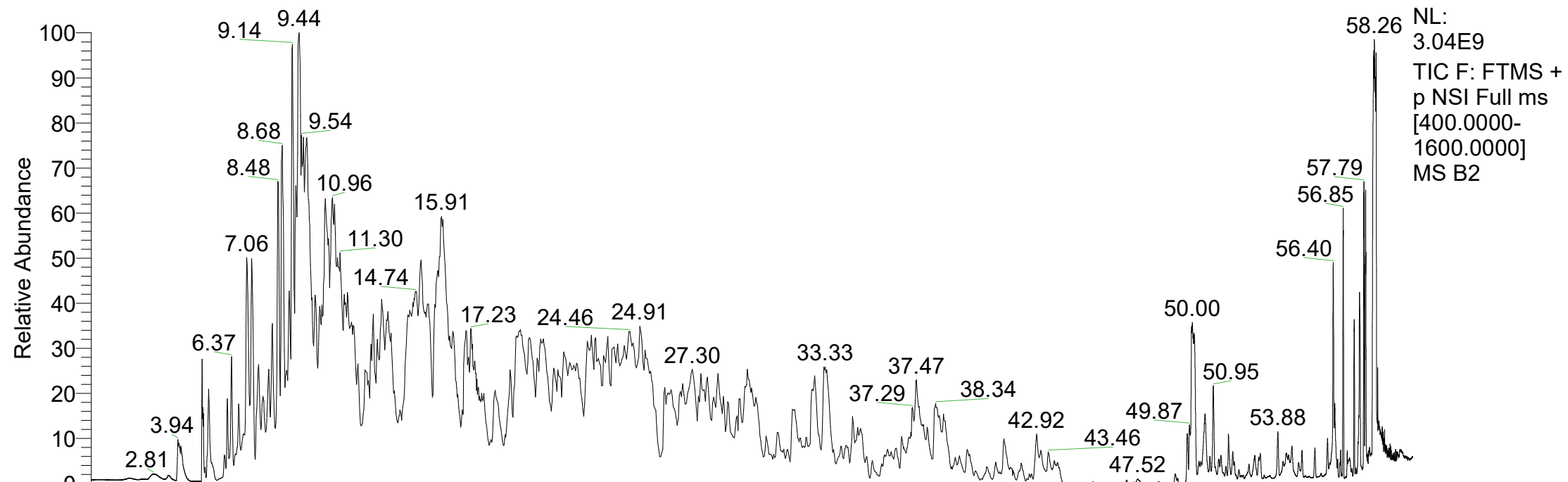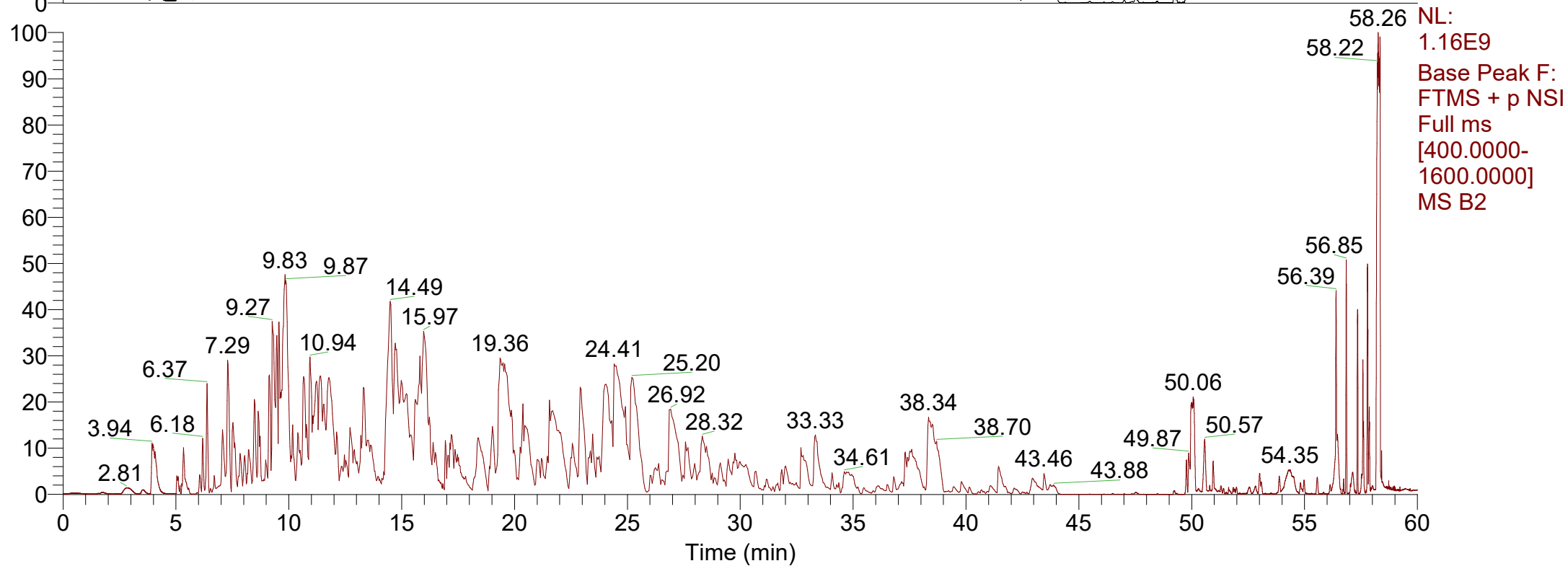

Supplement: Supplementary file 5 [file DataSheet5.pdf]

RT: 0.00 - 60.00

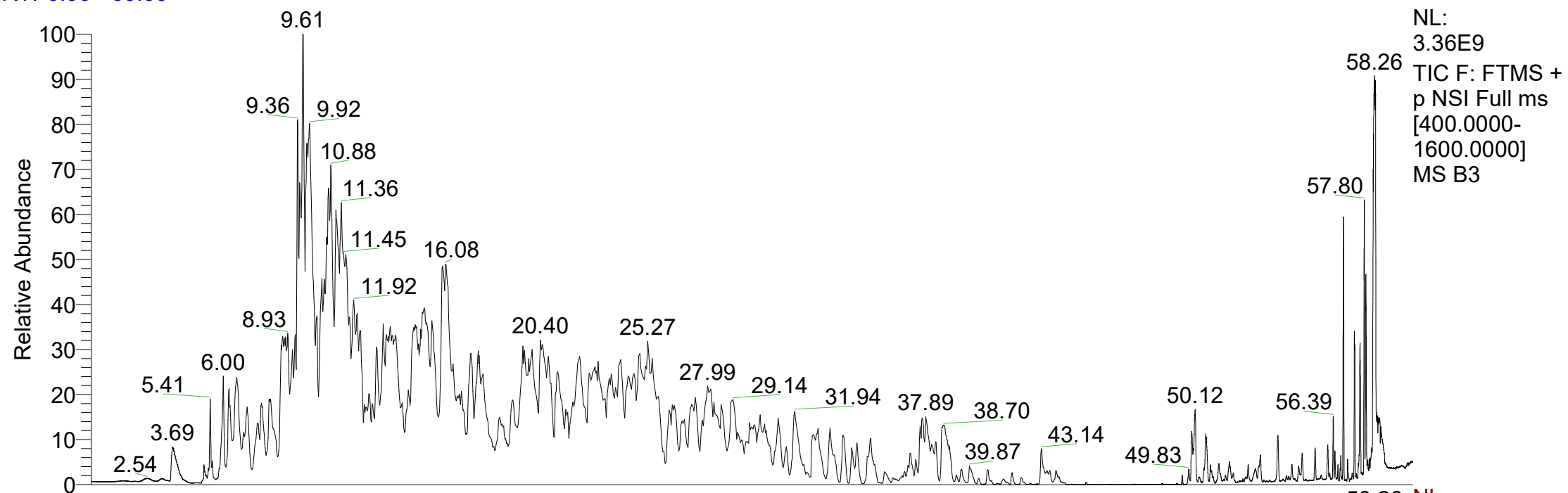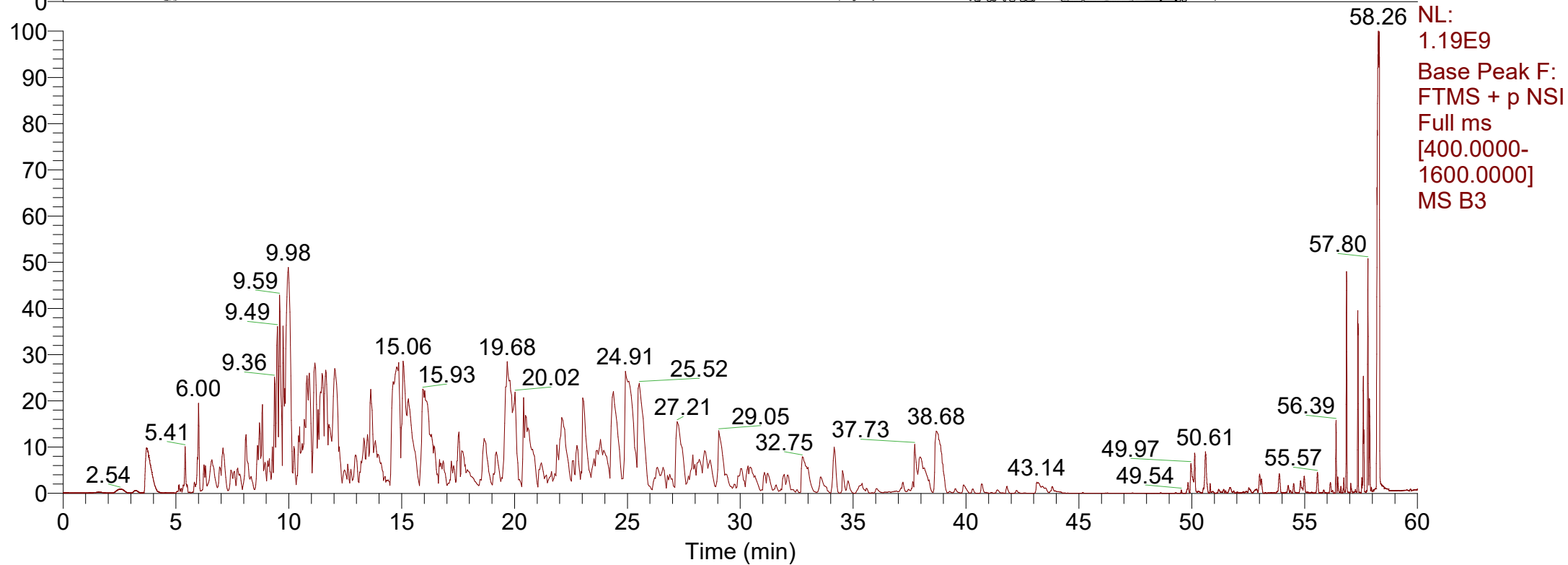

Supplement: Supplementary file 6 [file DataSheet6.pdf]

RT: 0.00 - 60.00

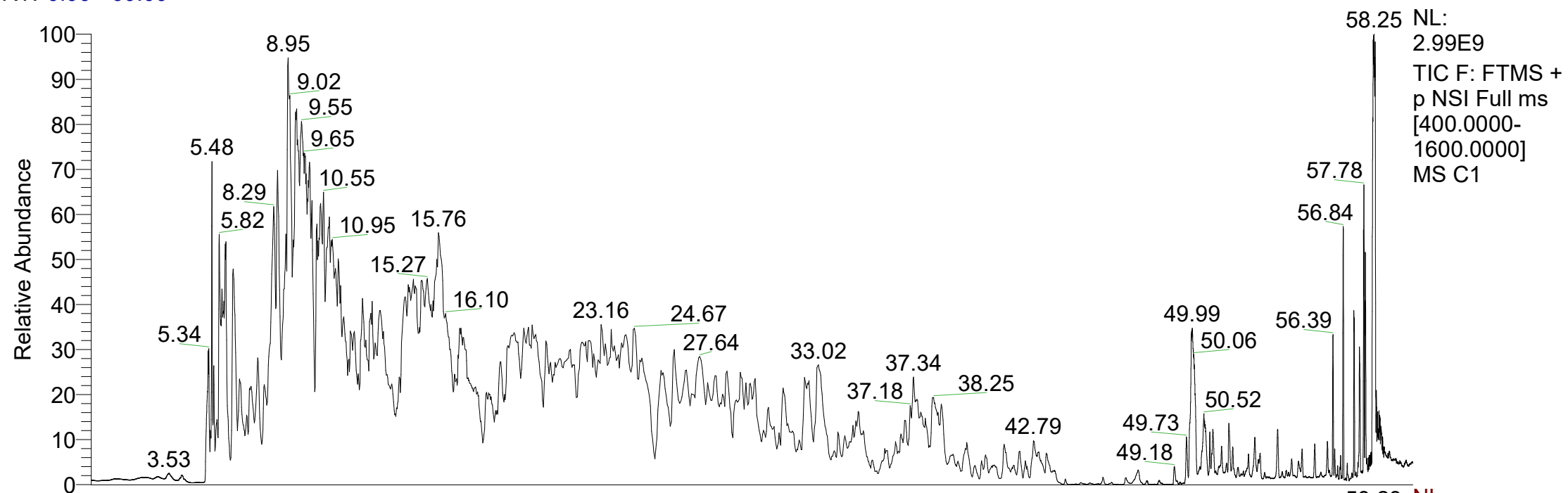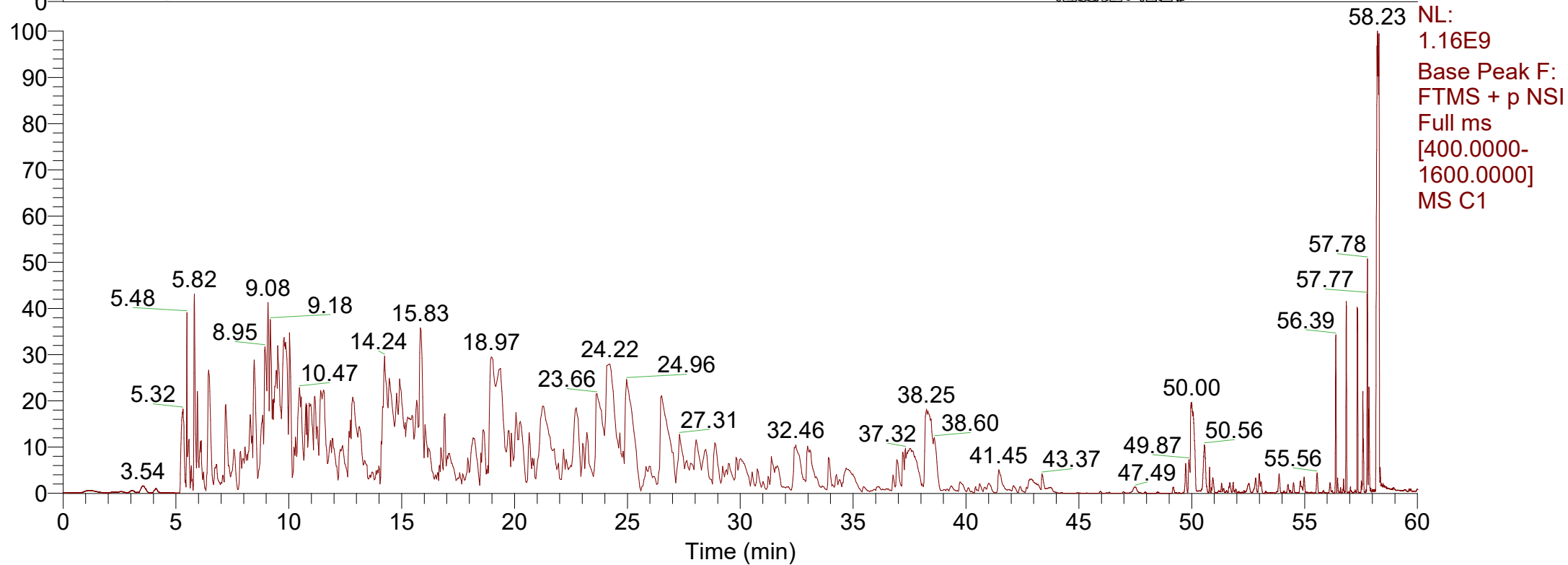

Supplement: Supplementary file 7 [file DataSheet7.pdf]

RT: 0.00 - 60.01

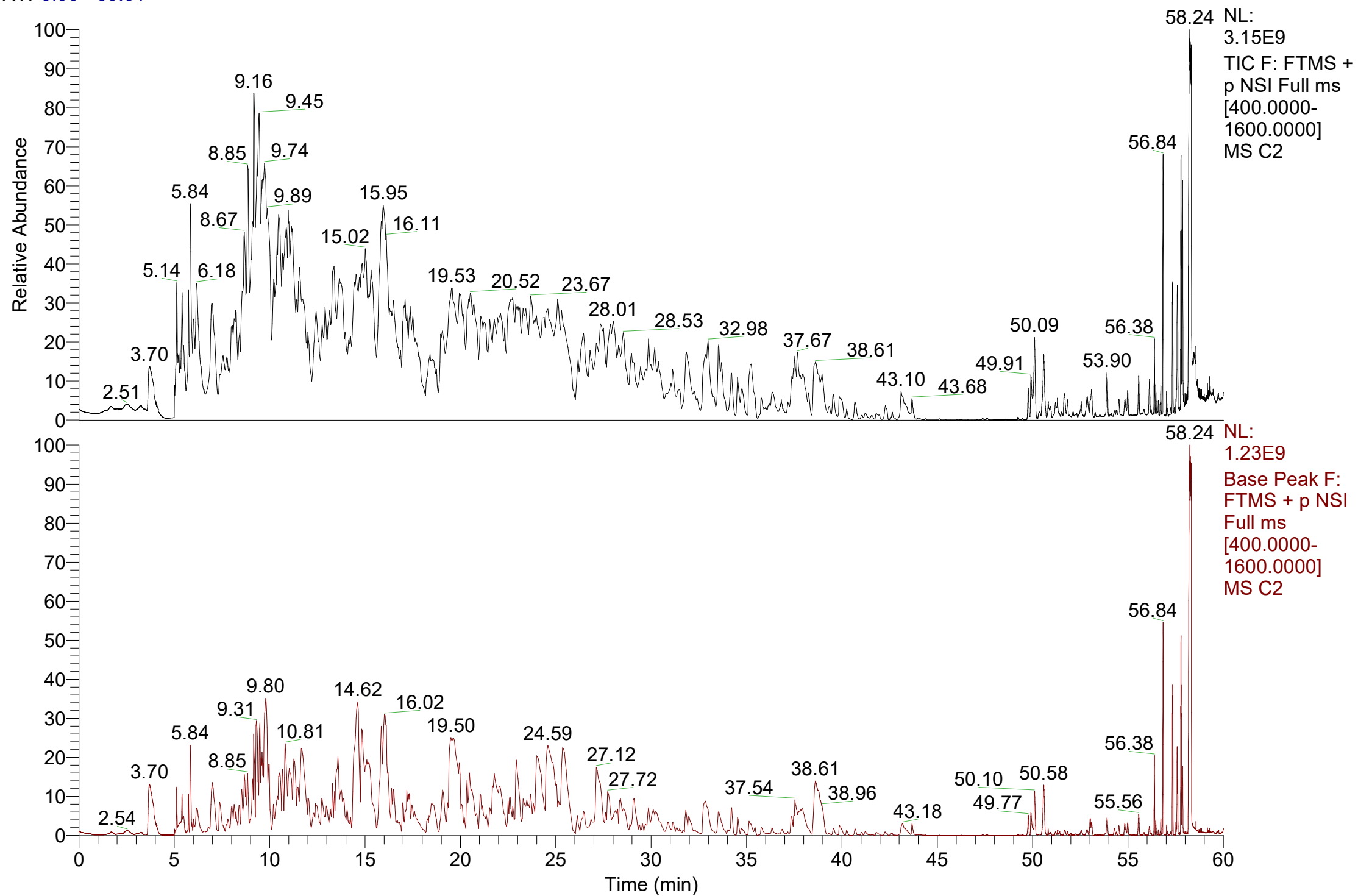

Supplement: Supplementary file 8 [file DataSheet8.pdf]

RT: 0.00 - 60.01

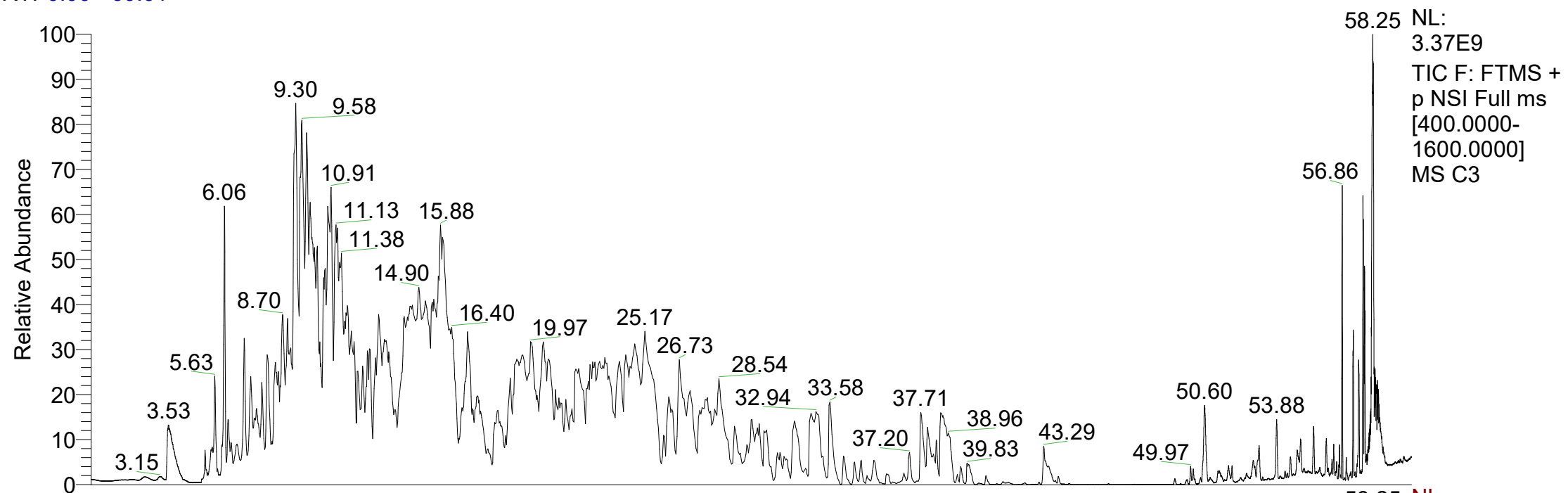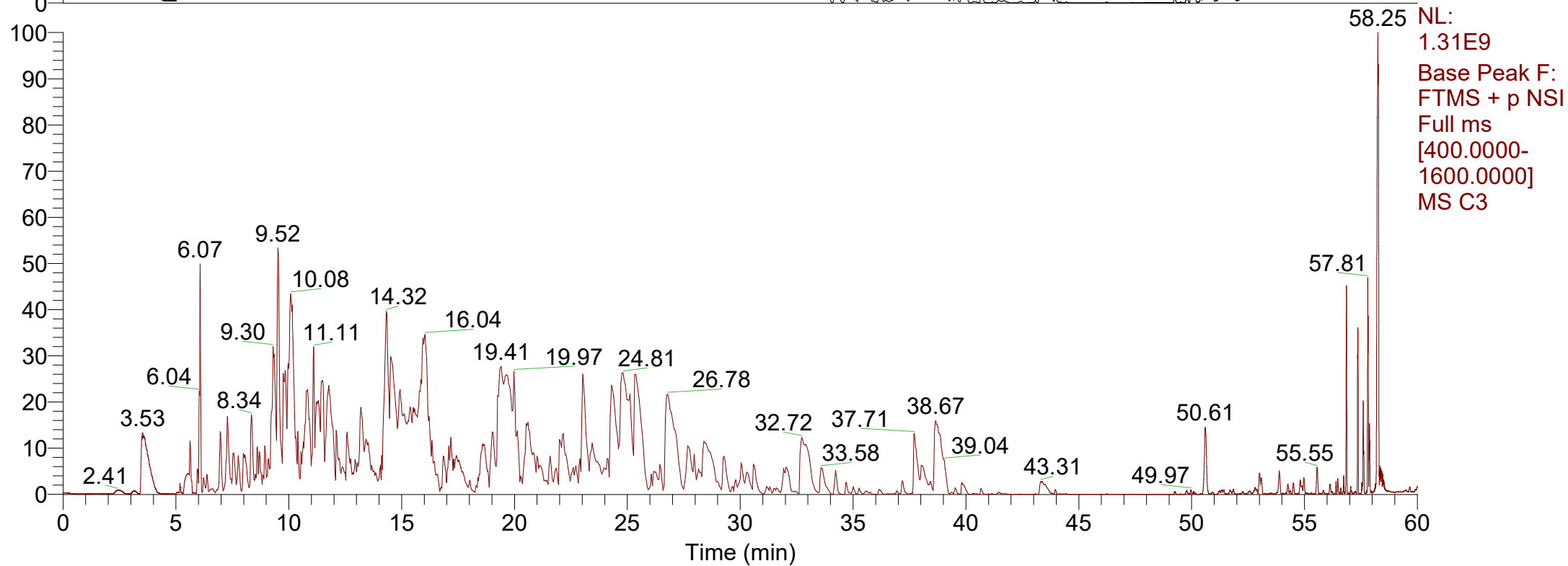

Supplement: Supplementary file 9 [file DataSheet9.pdf]

RT: 0.00 - 60.00

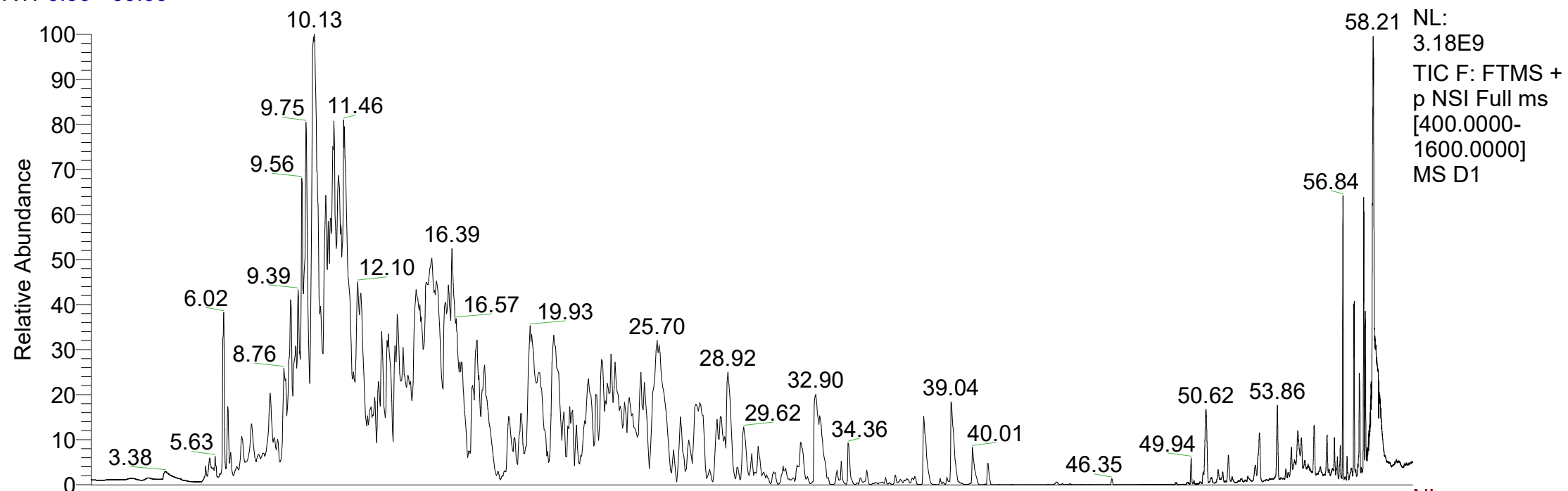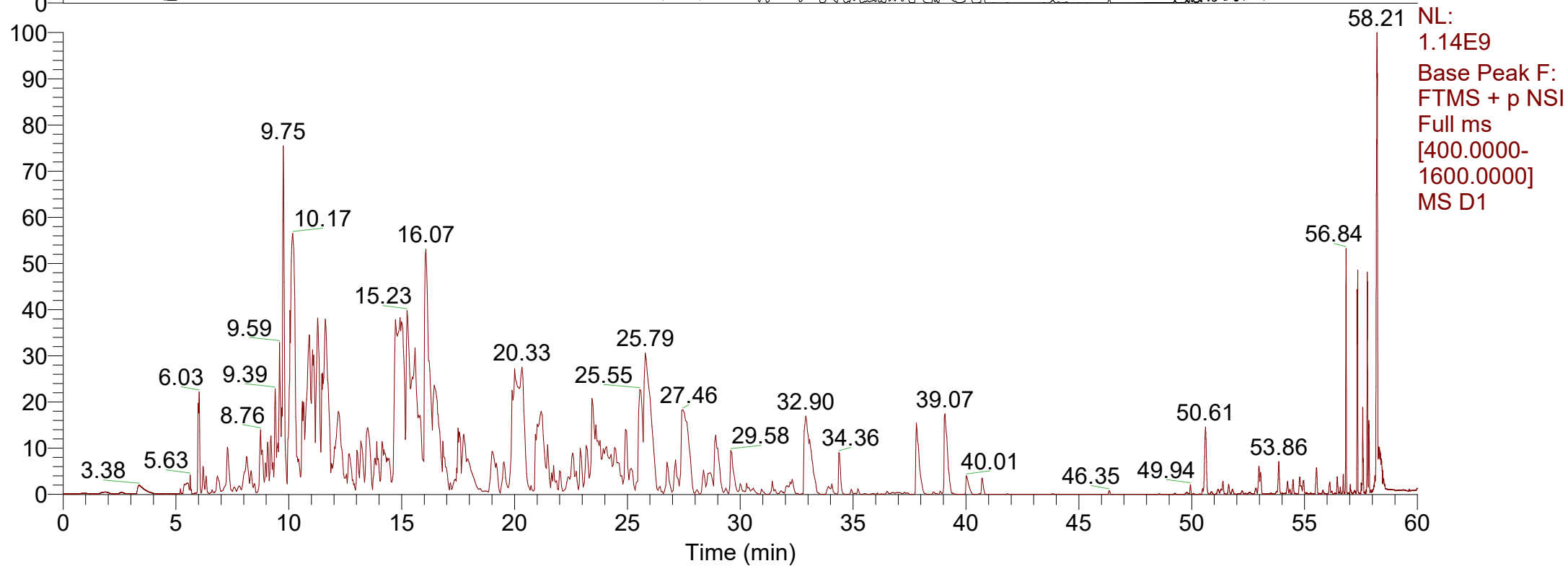

Supplement: Supplementary file 10 [file DataSheet10.pdf]

RT: 0.00 - 60.01

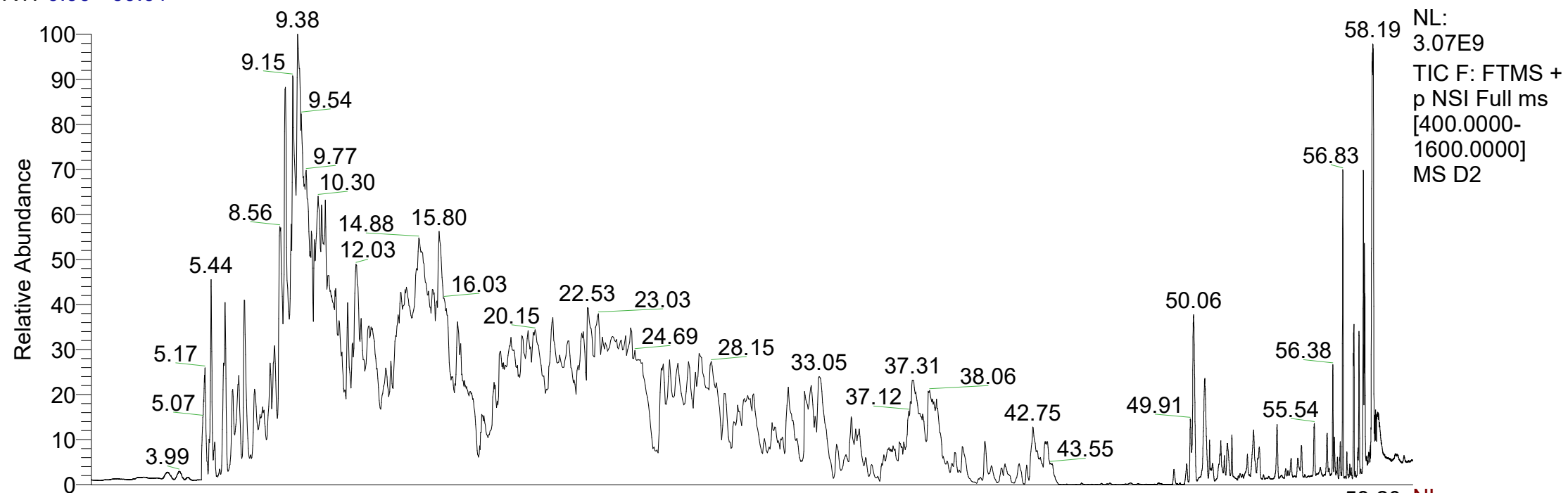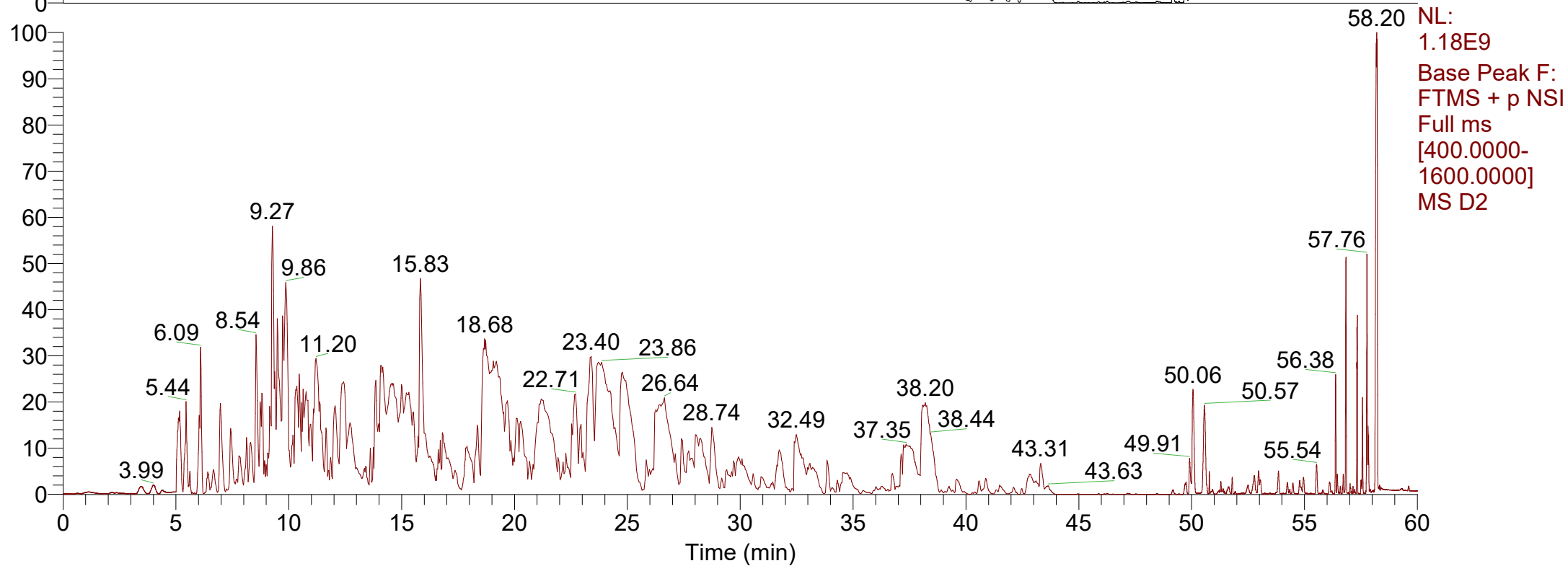

Supplement: Supplementary file 11 [file DataSheet11.pdf]

RT: 0.00 - 60.00

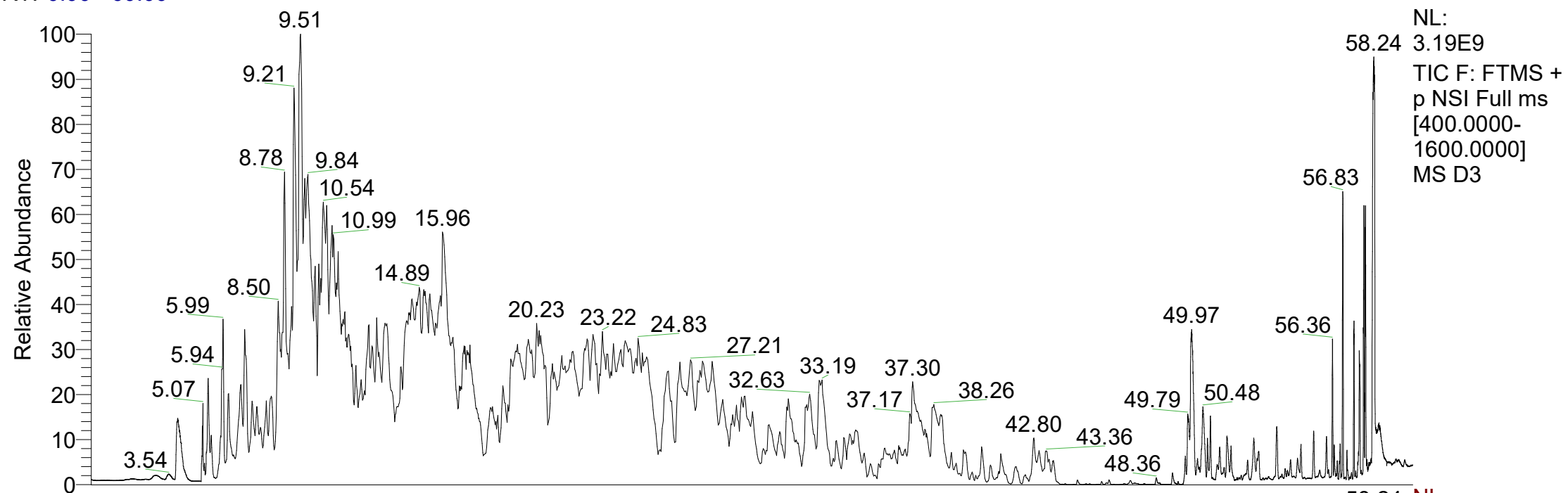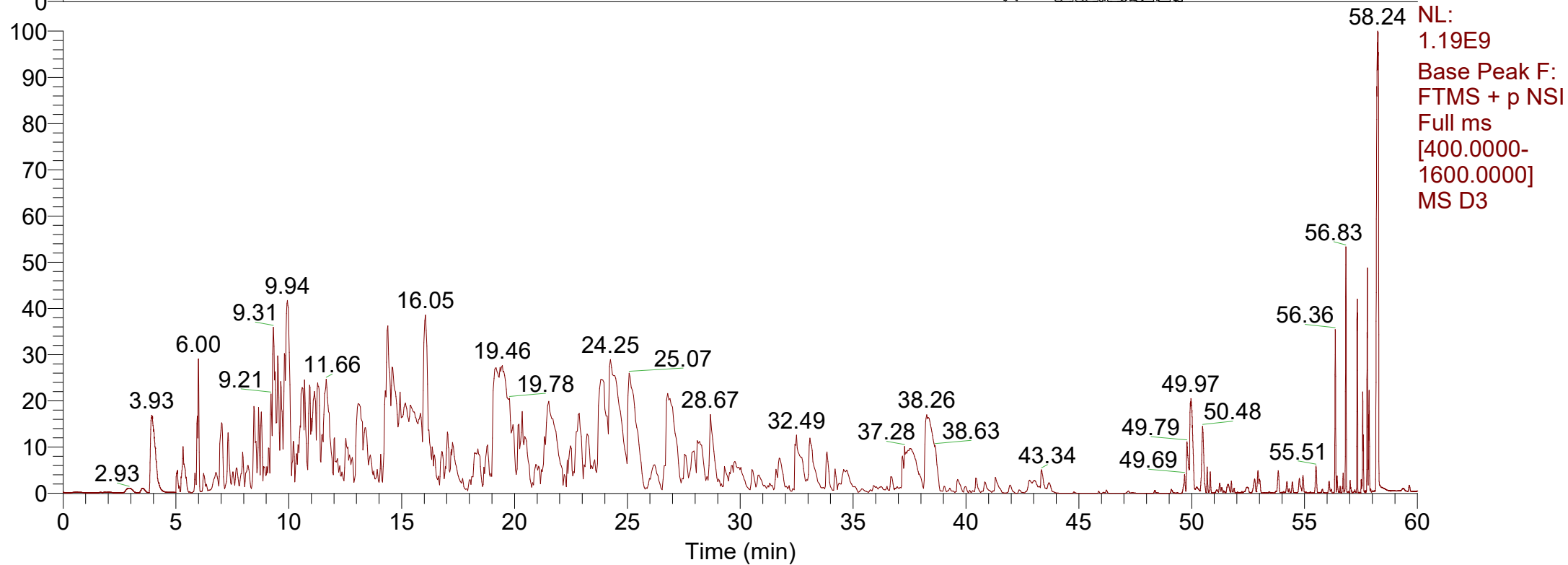

Supplement: Supplementary file 12 [file DataSheet12.pdf]

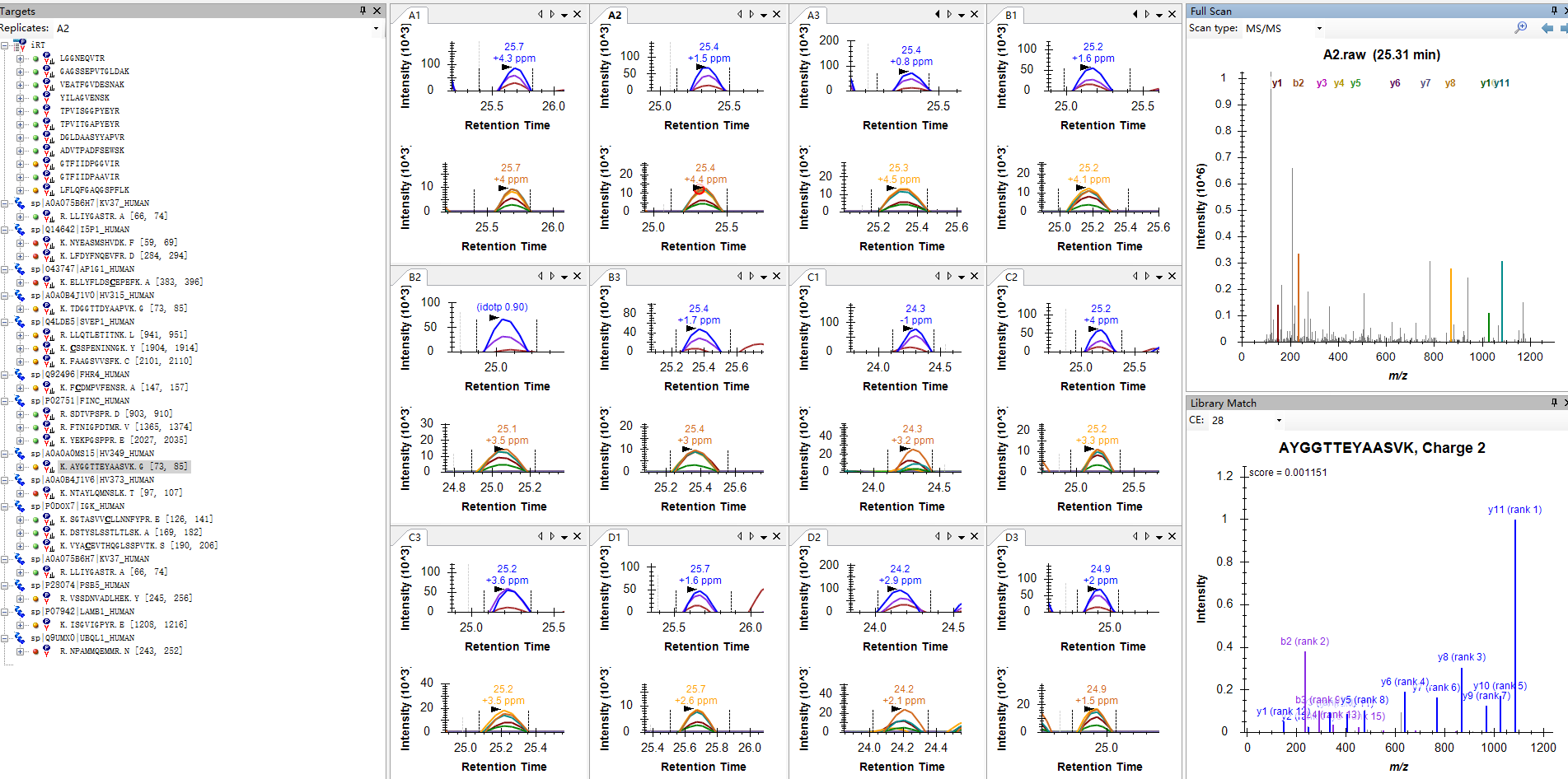

Supplement: Supplementary file 13 [file Image1.png]

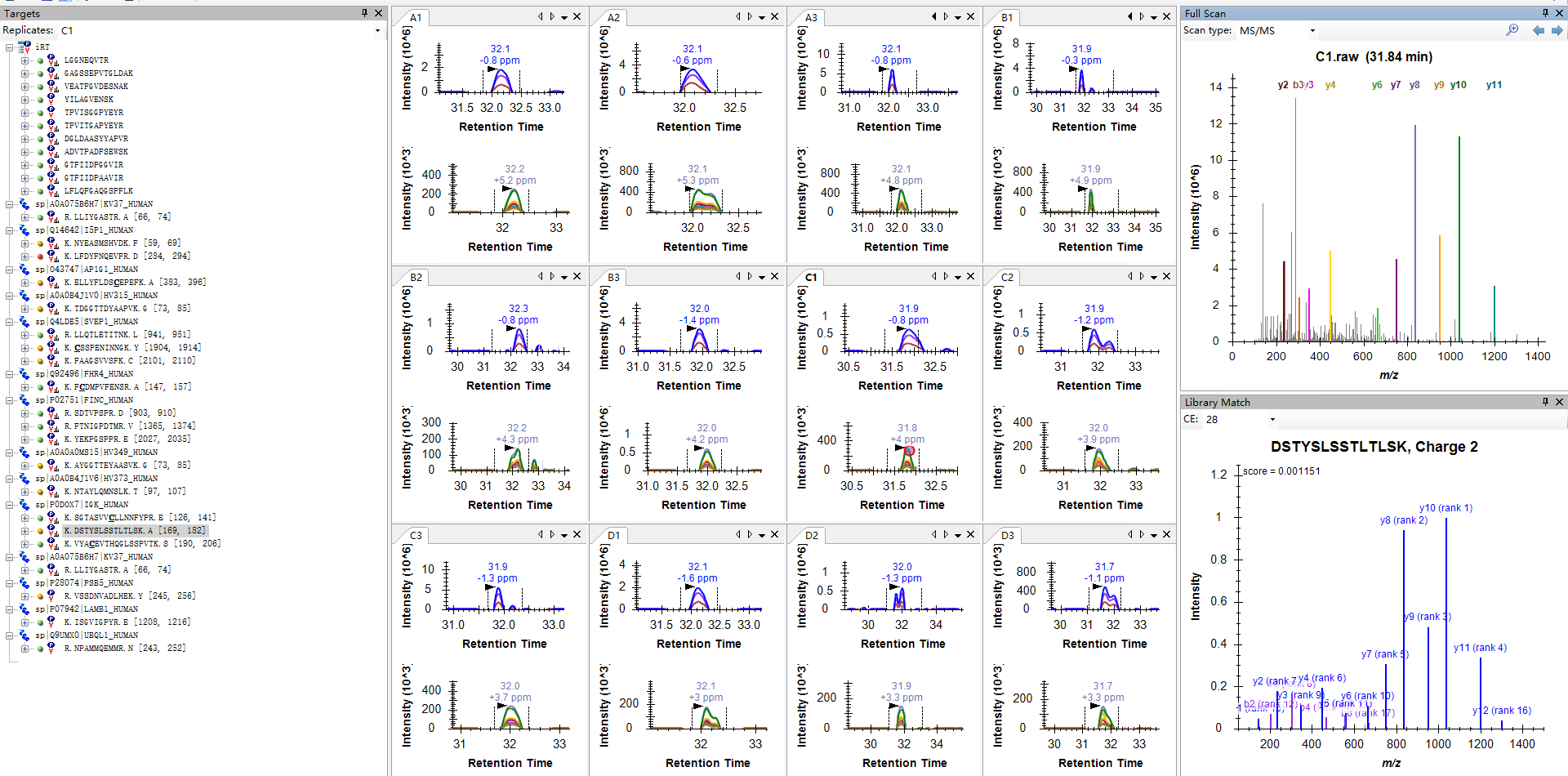

Supplement: Supplementary file 14 [file Image2.png]

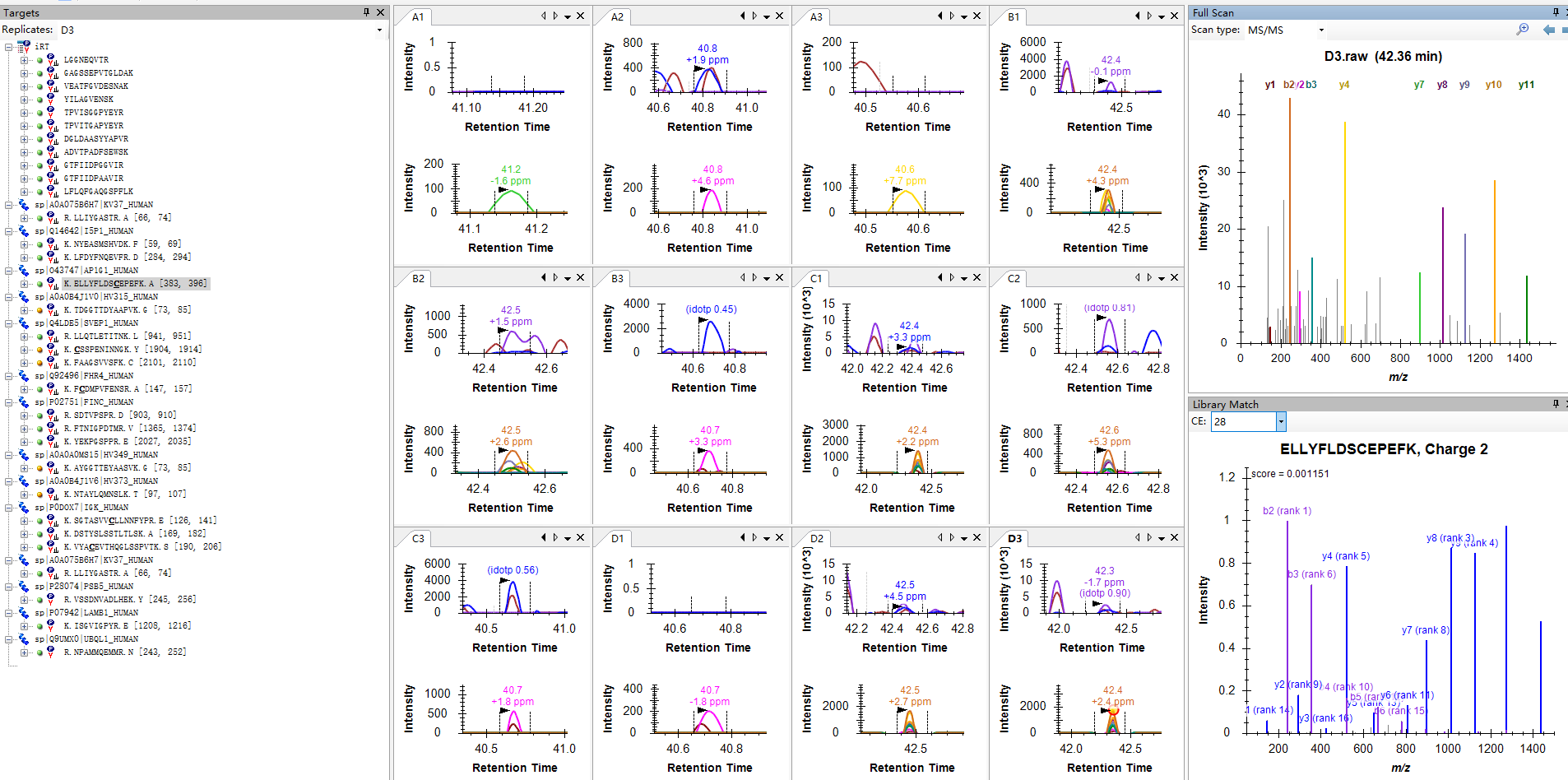

Supplement: Supplementary file 15 [file Image3.png]

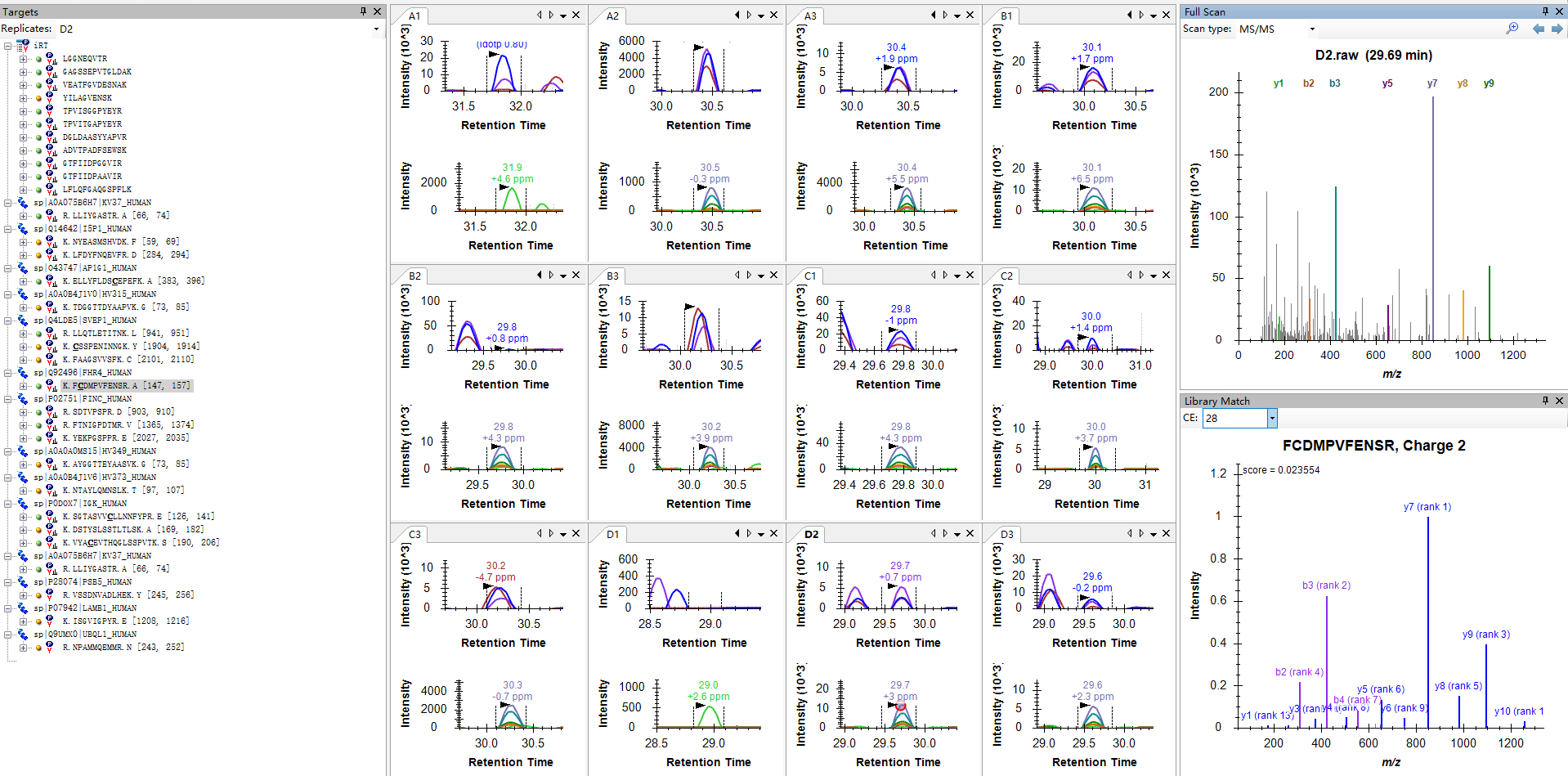

Supplement: Supplementary file 16 [file Image4.png]

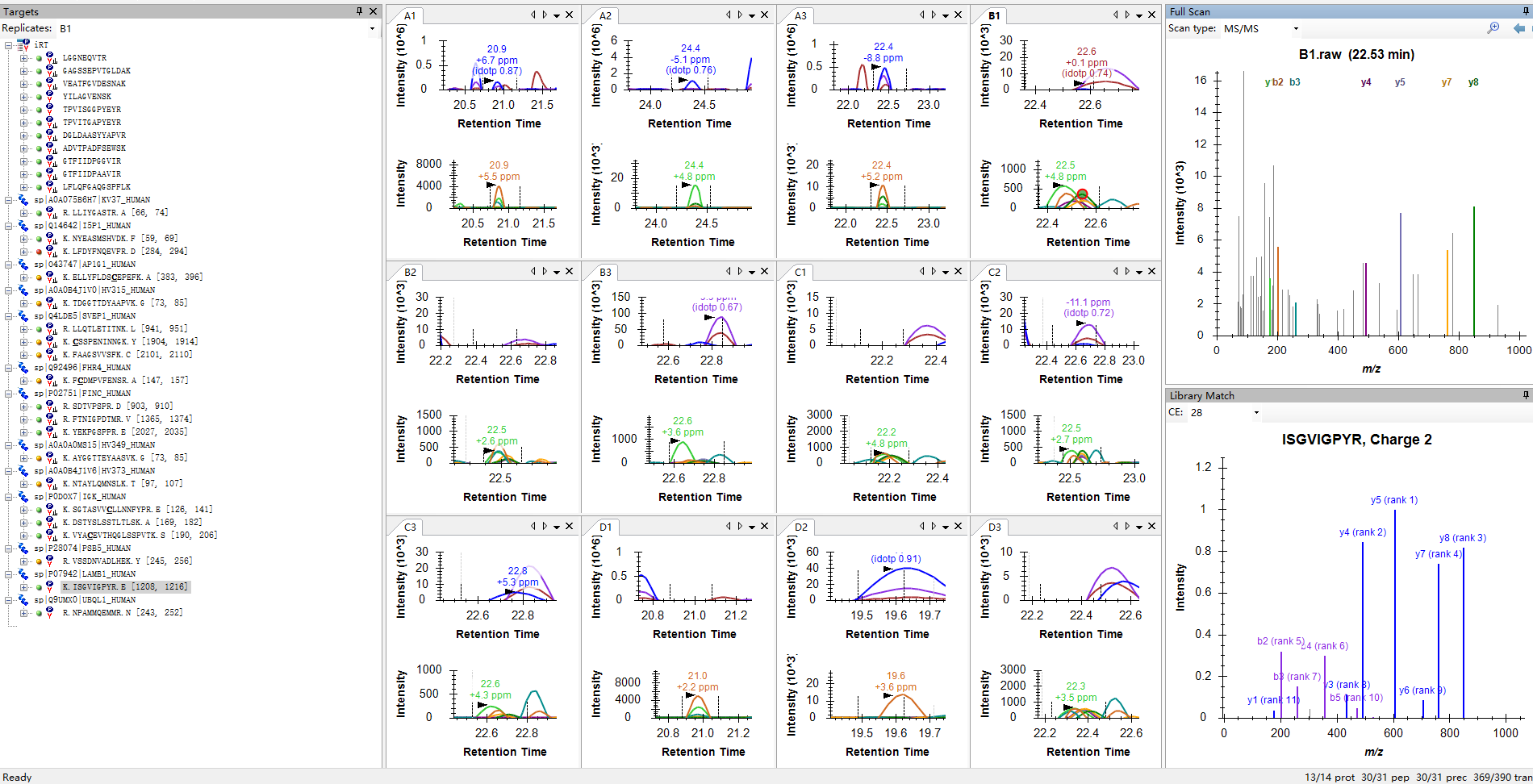

Supplement: Supplementary file 17 [file Image5.png]

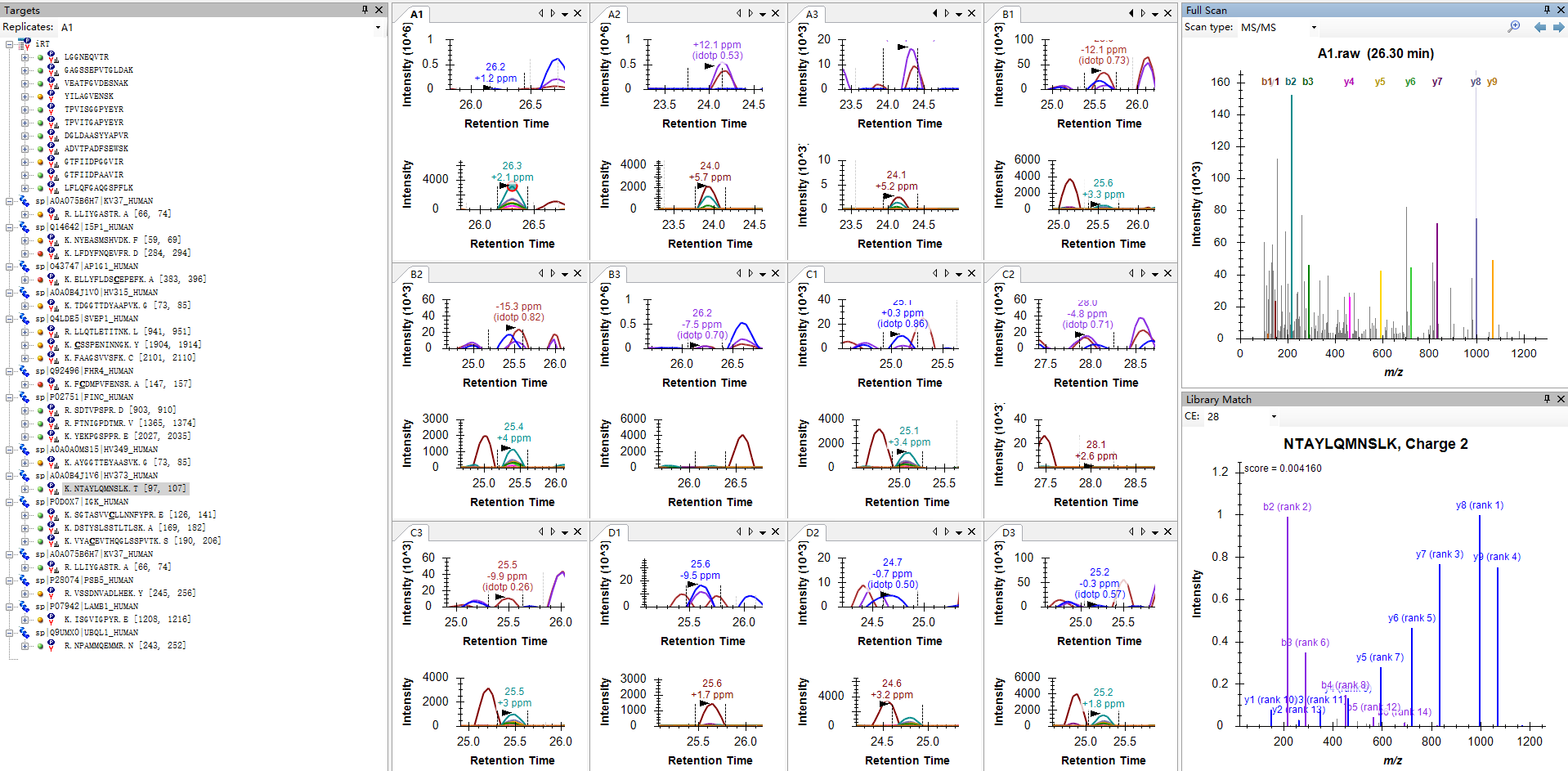

Supplement: Supplementary file 18 [file Image6.png]

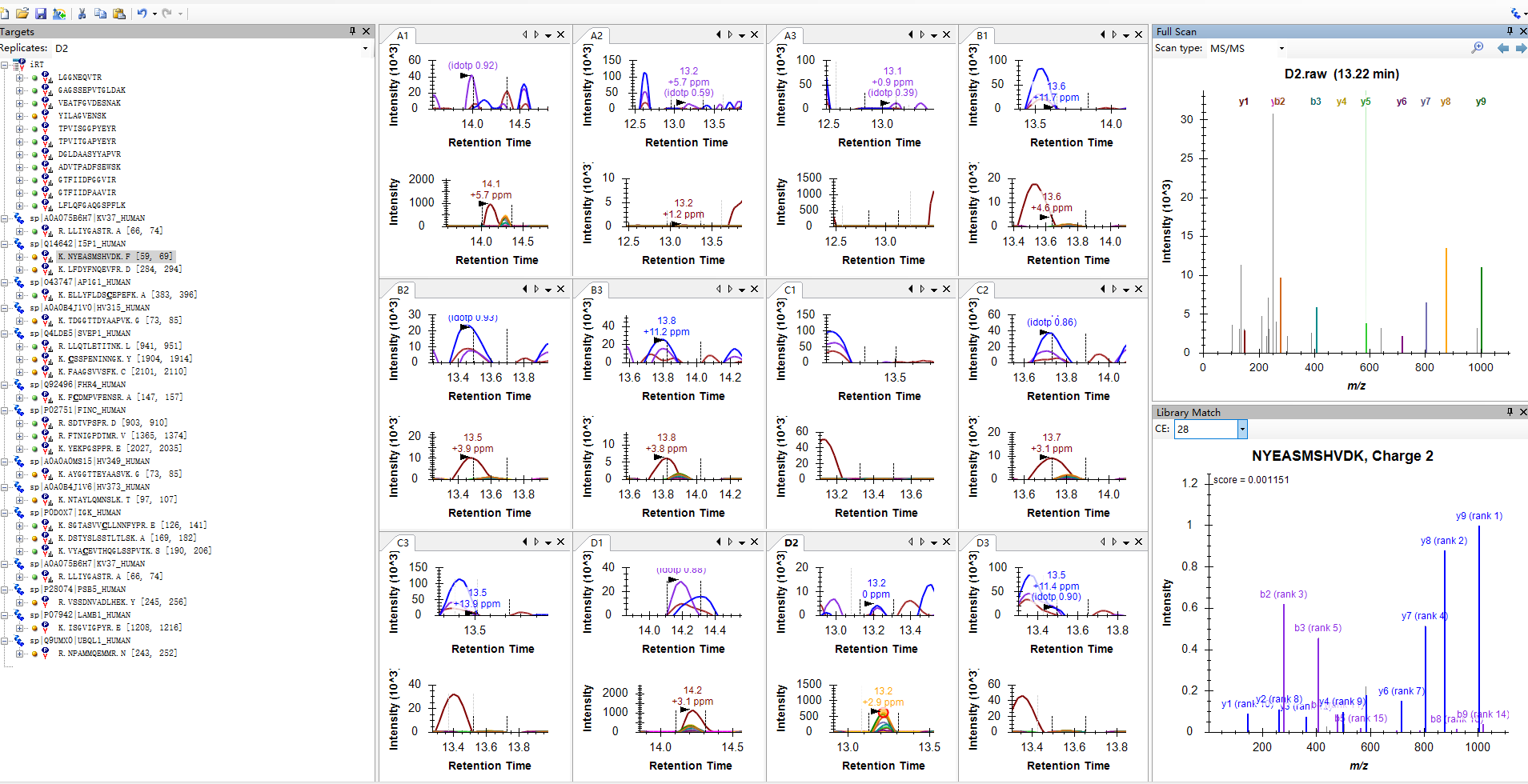

Supplement: Supplementary file 19 [file Image7.png]

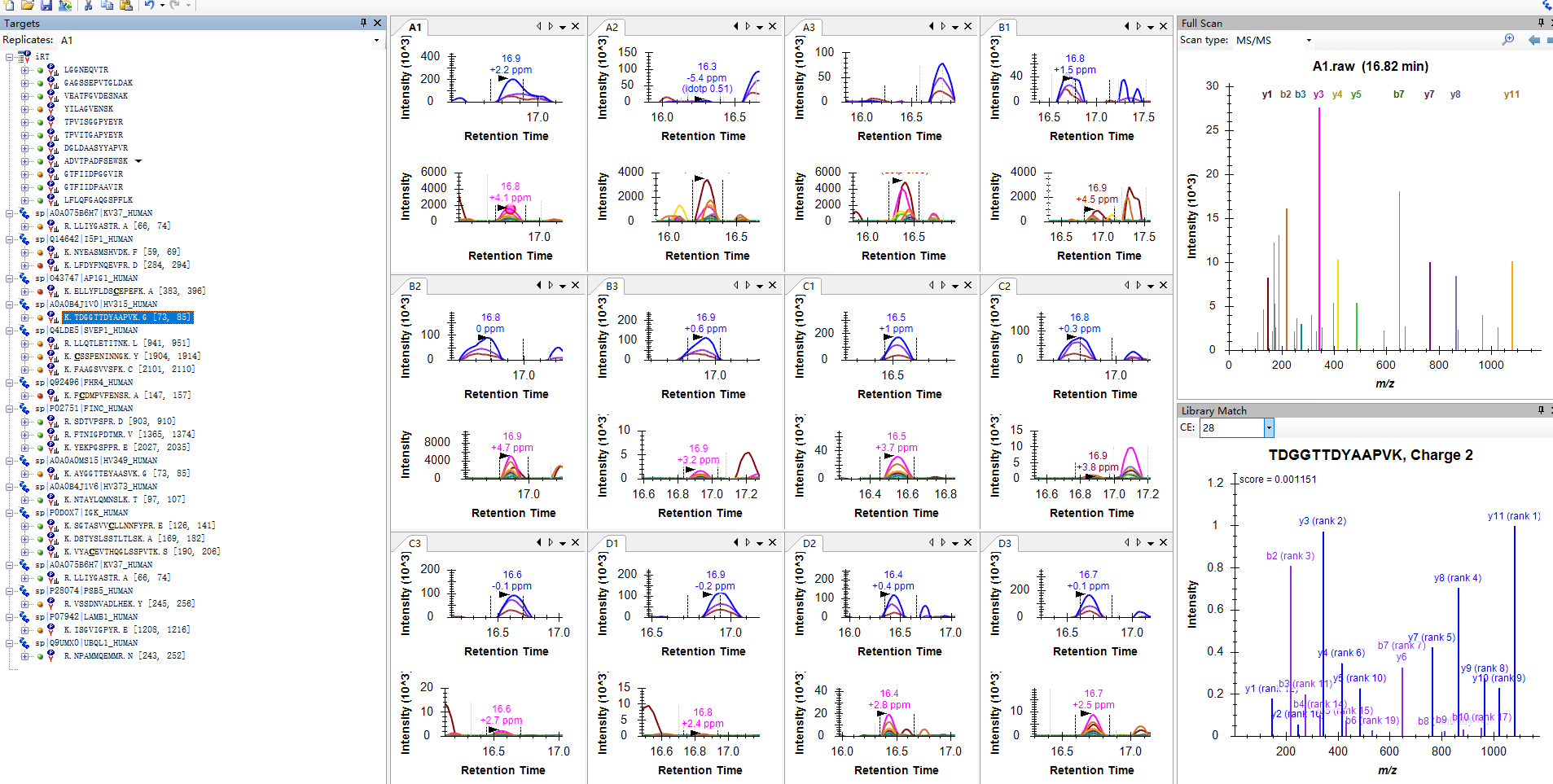

Supplement: Supplementary file 20 [file Image8.png]

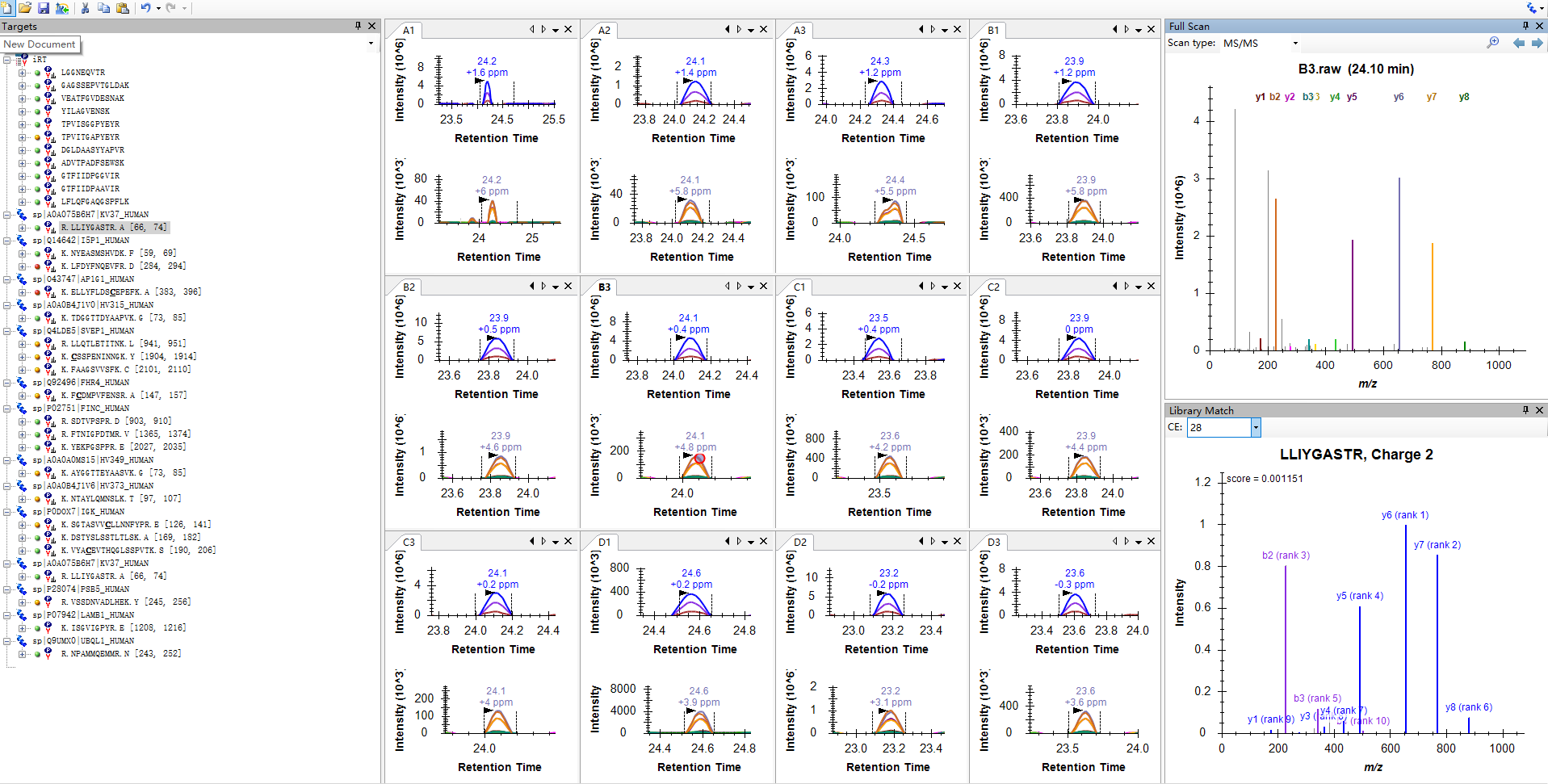

Supplement: Supplementary file 21 [file Image9.png]

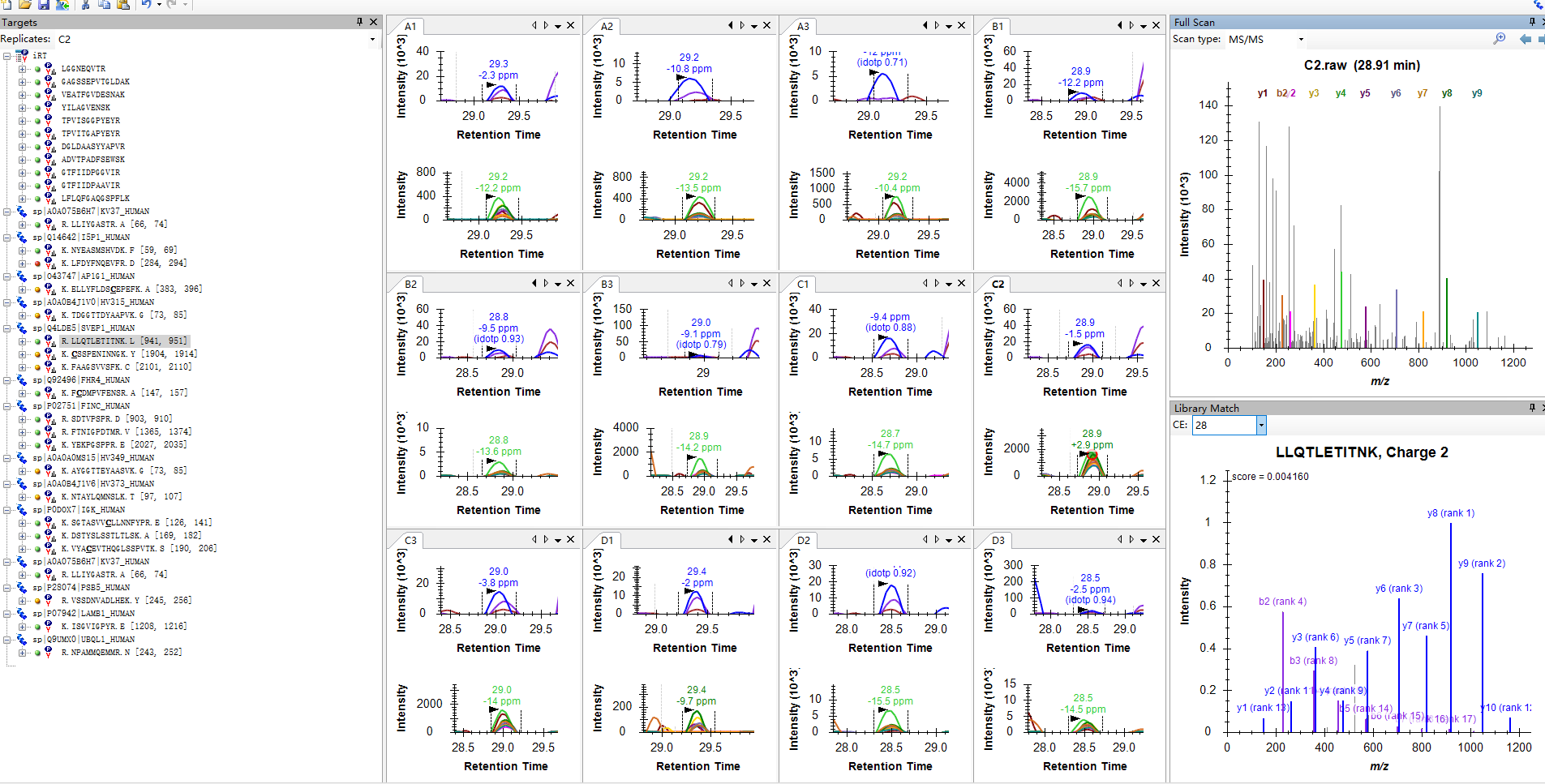

Supplement: Supplementary file 22 [file Image10.png]

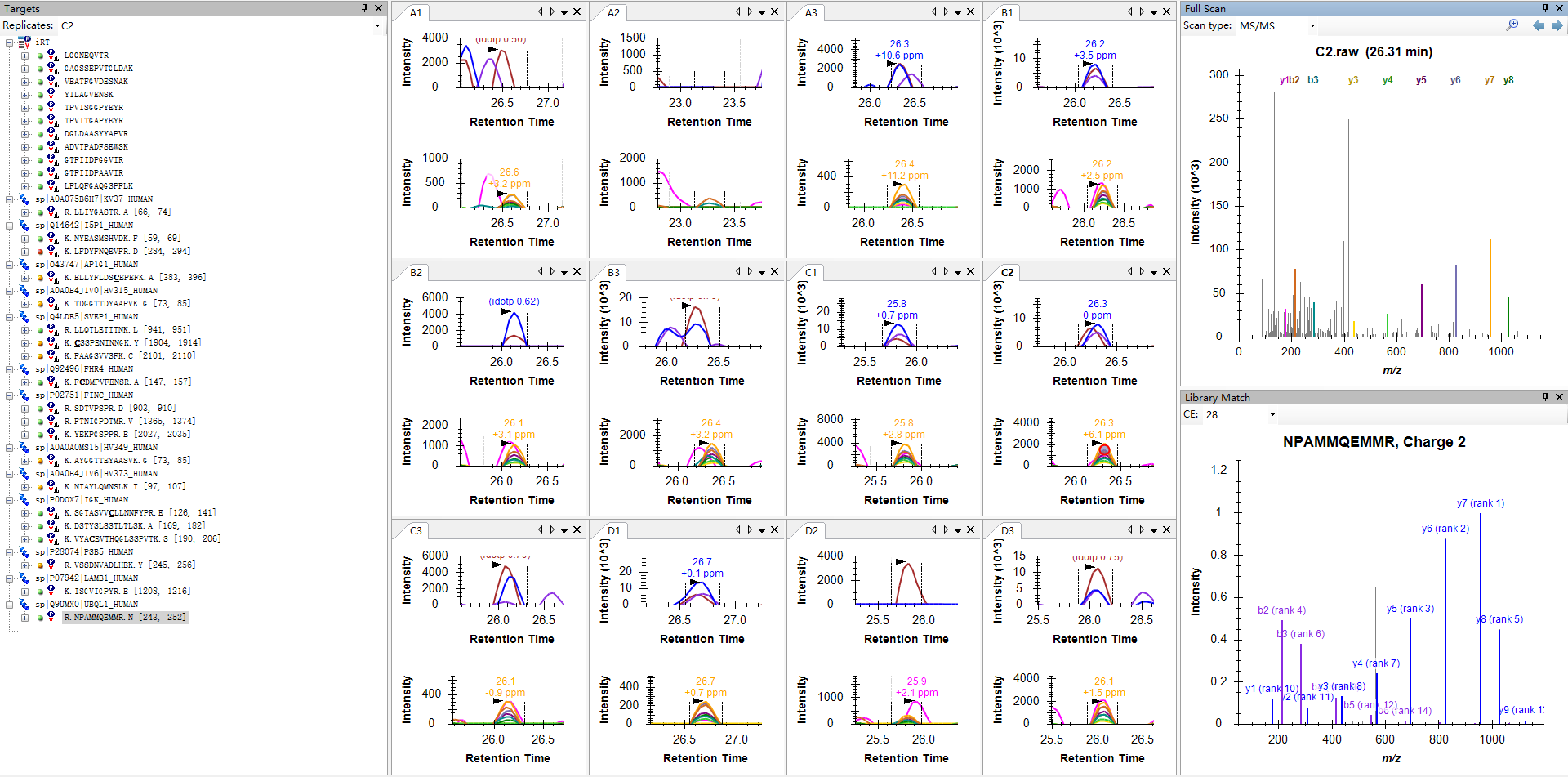

Supplement: Supplementary file 23 [file Image11.png]

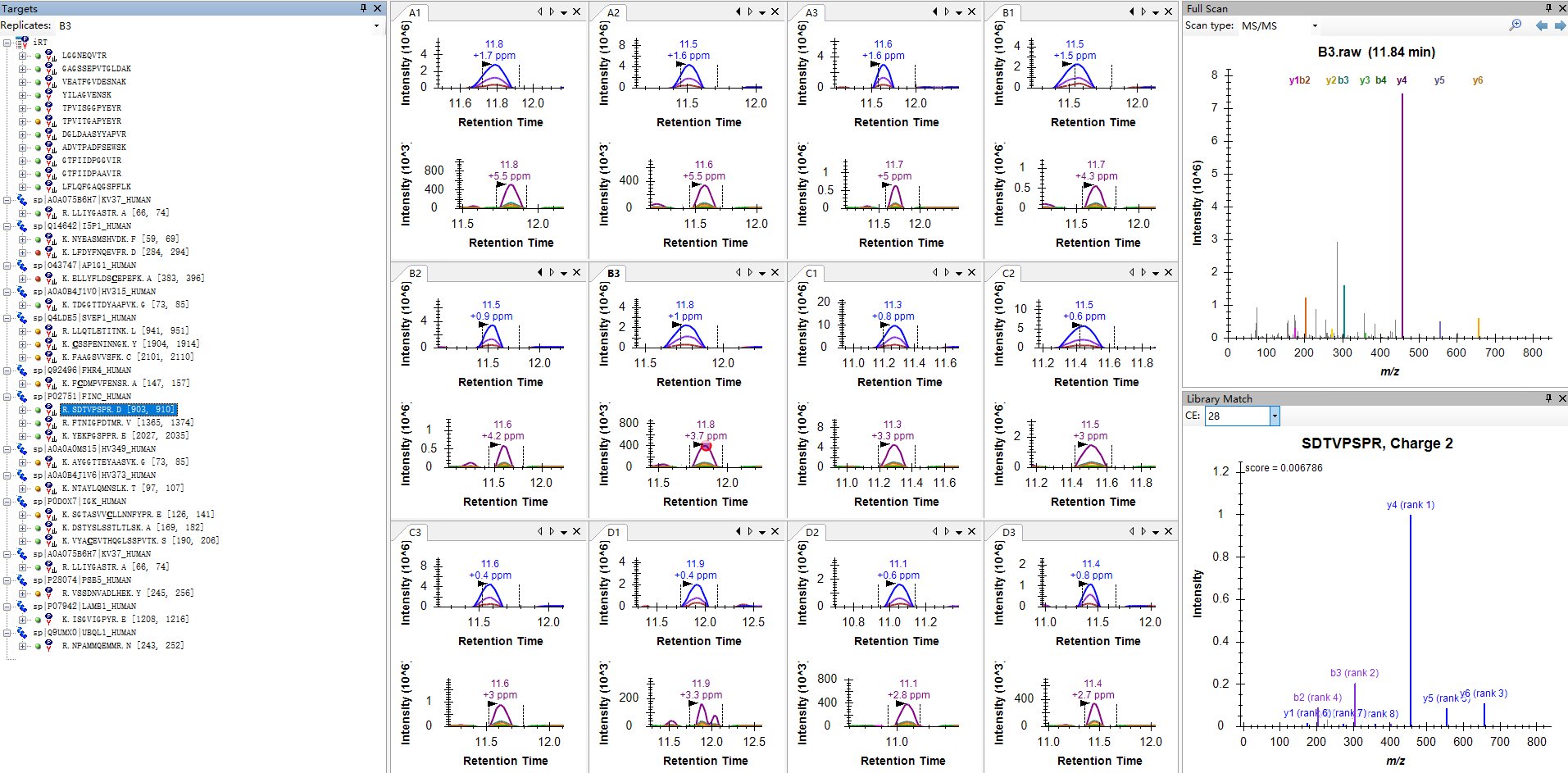

Supplement: Supplementary file 24 [file Image12.png]

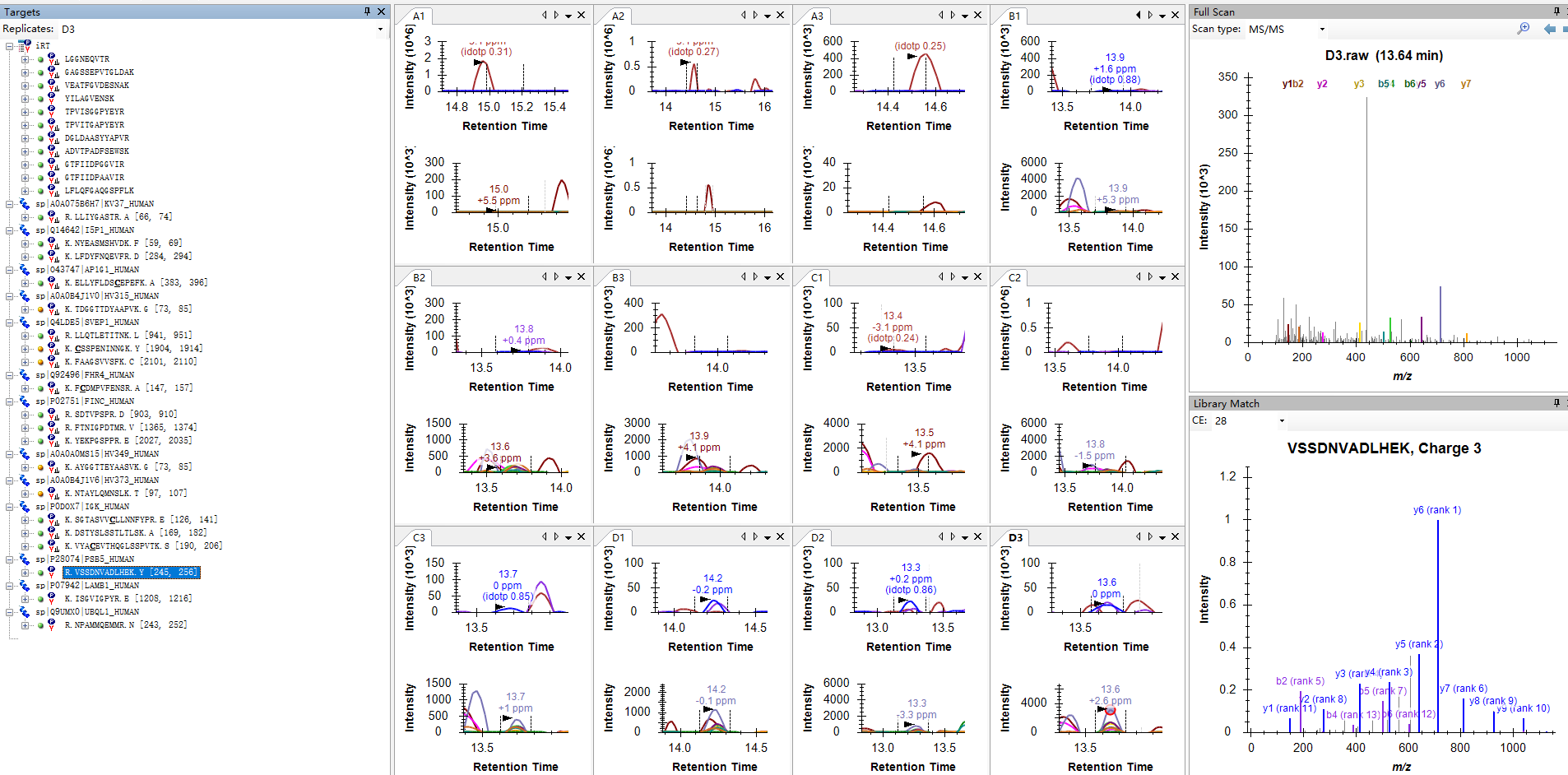

Supplement: Supplementary file 25 [file Image13.png]
